# Ptpn2 limits plasma cell fate and antiviral immunity by integrating B cell receptor and IFN-γ signals in B cells

**DOI:** 10.1126/sciadv.aec0162

**Published:** 2026-08-19

**Authors:** Ana Maria Hincapie, Alexandre Poirier, Isabelle Aubry, Philippe Aumont, Aanya Bhagrath, Noriko Uetani, Bianca Colalillo, Chenyue Wu, Benoit Charbonneau, Stephanie Bussieres-Marmen, Belma M. Abidin, Javier M. Di Noia, Judith N. Mandl, Silvia M. Vidal, Jorg H. Fritz, Michel L. Tremblay

**Affiliations:** ^1^ Department of Biochemistry, McGill University, Montreal, QC, Canada.; ^2^ Goodman Cancer Institute, McGill University, Montreal, QC, Canada.; ^3^ Department of Physiology, McGill University, Montreal, QC, Canada.; ^4^ McGill University Research Centre on Complex Traits, Montreal, QC, Canada.; ^5^ Department of Microbiology and Immunology, Montreal, QC, Canada.; ^6^ Department of Human Genetics, McGill University, Montreal, QC, Canada.; ^7^ Institut de Recherches Cliniques de Montréal, Montreal, QC, Canada.

## Abstract

Antigen-specific humoral responses are critical for long-term protection against infectious diseases, yet the mechanisms that regulate B cell differentiation and antibody production remain incompletely defined. Here, we identify the Protein Tyrosine Phosphatase Nonreceptor Type 2 (Ptpn2) as a B cell–intrinsic regulator of plasma cell fate and isotype switching. Using a B cell–specific Ptpn2 knockout mouse model, we show that Ptpn2 restrains both B cell receptor and interferon-γ (IFN-γ) signaling by directly dephosphorylating Lyn, STAT1, and STAT3. Loss of Ptpn2 leads to hyperactivation of these two signaling pathways, resulting in transcriptional reprogramming that promotes plasma cell differentiation and increased IFN-γ–driven antibody production. Functionally, Ptpn2-deficient mice generated enhanced primary antiviral antibody responses following influenza infection and elevated virus-specific and neutralizing titers upon recall without compromising affinity. These findings identify Ptpn2 as a key intracellular checkpoint that integrates antigenic and inflammatory cues to regulate humoral immunity, with potential implications for enhancing vaccine-induced protective immunity.

## INTRODUCTION

B cells are central to adaptive immunity, producing antibodies that recognize a broad range of antigens. These antibodies can bind to and neutralize diverse pathogens, such as bacteria and viruses, thus preventing infection and contributing to long-term immunity. Mature B cells exist in two major subsets, marginal zone (MZ) and follicular (Fo) B cells, each carrying out distinct roles during infection. MZ B cells can rapidly differentiate into short-lived antibody-secreting cells that help control the initial stages of an infection (*1*, *2*). On the other hand, Fo B cells respond at later stages of the infection and differentiate into effector B cells that produce antibodies with high affinity for invading pathogens. Upon activation, Fo B cells migrate into germinal centers (GCs), where they undergo affinity maturation and somatic hypermutation, two processes that optimize antibody specificity and affinity (*3*, *4*). Activation of B cells can be triggered through multiple pathways, depending on the context of antigen encounter. The primary signal is delivered through the B cell receptor (BCR) upon direct antigen recognition, initiating intracellular signaling cascades that promote proliferation and differentiation (*5*, *6*). BCR signaling is often accompanied by secondary inputs, either from interactions with other immune cells via direct cell-surface receptor contact or through the secretion of pro-inflammatory cytokines (*7*, *8*). Following the GC reaction, selected B cells can differentiate into long-lived antibody-secreting cells known as plasma cells (PCs) or into memory B cells, both of which are responsible for long-lasting humoral immunity (*9*–*11*).

The differentiation of B cells into PCs is a tightly regulated process governed by a complex network of transcription factors (TFs) and cytokine signals. Key TFs such as Blimp-1 (*Prdm1*), IRF4, and XBP1 drive the PC program by repressing the GC B cell transcriptional network and promoting the machinery necessary for antibody secretion (*12*–*14*). Cytokines in the microenvironment, such as interferon-γ (IFN-γ), further shape this process by enhancing class-switch recombination to specific isotypes, allowing the immune system to tailor the humoral response (*15*, *16*). Understanding how PC differentiation is regulated is essential for developing strategies to modulate B cell responses following vaccination or during autoimmunity.

T cell protein tyrosine phosphatase [TC-PTP; protein tyrosine phosphatase nonreceptor type 2 (PTPN2)] is a known negative regulator of cytokine signaling, primarily through its ability to dephosphorylate Janus kinase (JAK) and signal transducer and activator of transcription (STAT) family proteins, which are key signaling mediators of interferons and interleukins (*17*–*20*). In addition, Ptpn2 also targets Src-family kinases, positioning it as a central regulator of immune cell signaling pathways (*21*, *22*). Consequently, this phosphatase has been described to regulate the development and activation of several populations of immune cells such as T cells, macrophages, and dendritic cells (*22*–*28*). In line with this, mutations in the *PTPN2* gene have been linked to the development of autoimmune diseases such as type 1 diabetes and rheumatoid arthritis (*29*–*32*). Notably, several of the patients carrying *PTPN2* mutations exhibit manifestations of B cell–driven autoimmunity, such as the presence of circulating autoantibodies (*32*).

Mouse models of *Ptpn2* deficiency have begun to provide mechanistic insight into how this phosphatase regulates B cell tolerance and activation (*25*, *33*). Global deletion of *Ptpn2* leads to a block in development at the pre-B cell stage in the bone marrow because of an inability to respond to interleukin-7 (IL-7), resulting from STAT1 hyperphosphorylation (*33*, *34*). More recently, hematopoietic deletion of Ptpn2 has been shown to drive systemic autoimmunity and insulitis, resulting from the aberrant activation of B cells and T follicular helper cells (T_FH_ cells), a phenotype mediated by enhanced STAT3 hyperphosphorylation in response to IL-21 (*35*). Recent work demonstrated that loss of Ptpn2 in B cells promotes immune activation and the emergence of autoreactive features, including increased autoantibody production and organ immune infiltration (*36*). Together, these findings point to a critical role for Ptpn2 in B cell biology, both during development and in the periphery. However, how Ptpn2 regulates B cell activation and humoral immunity in response to immunological challenge remains unclear. In this study, we address this gap by generating a mouse model in which Ptpn2 deficiency is restricted to B cells. Our work reveals a role for this phosphatase in limiting PC differentiation, IFN-γ–driven isotype switching, and antibody secretion. We show that abolishing Ptpn2 expression in B cells drives increased antibody responses against influenza A virus infection, highlighting Ptpn2 as a potential target for enhancing vaccine-induced immunity.

## RESULTS

### Deletion of Ptpn2 in B cells leads to reduced MZ B cell frequencies and responses

**Fig. 1.**
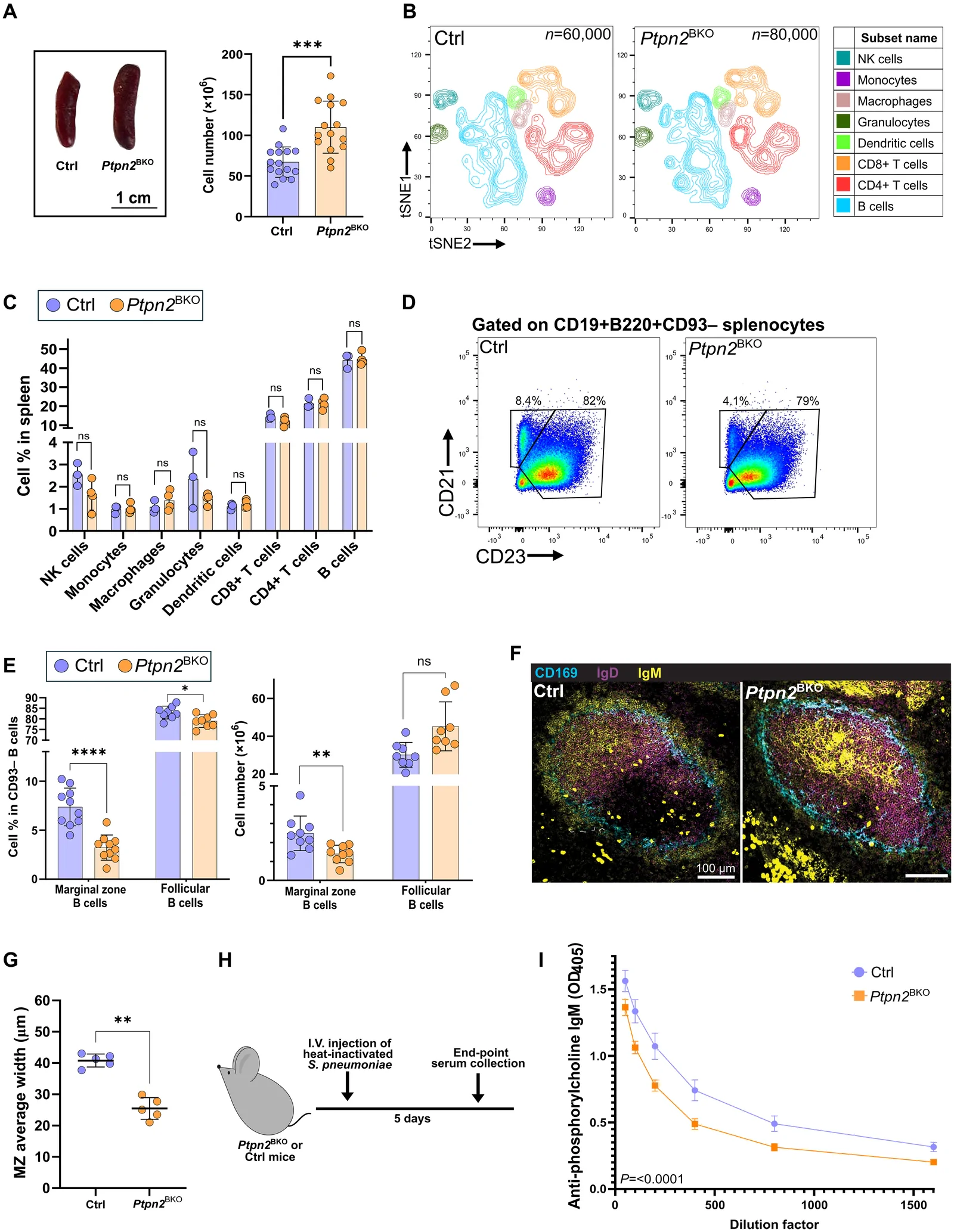
*Ptpn2* deficiency in B cells leads to splenomegaly and reduction of MZ B cells. All panels compare Ptpn2^fl/fl^ MB1-cre (*Ptpn2*^BKO^) versus Ptpn2^wt/wt^MB1-cre (Ctrl) mice. (**A**) Quantification of splenocyte numbers and representative photograph of spleens of 10-week-old mice of the indicated genotypes. (**B**) t-SNE clustering of splenic immune cell populations. Cell identities were assigned on the basis of surface marker expression (table S2). Total cell numbers used for each plot are indicated. NK cells, natural killer cells. (**C**) Frequencies of splenic immune cell subsets. Data are representative of at least two independent experiments. (**D**) Representative flow cytometry plots of splenic CD19^+^B220^+^CD93^−^CD21/35^hi^CD23^lo^ MZ and CD19^+^B220^+^CD93^−^CD21/35^lo^CD23^hi^ Fo B cells. (**E**) Proportions and absolute cell numbers of splenic MZ and Fo B cells. Data are representative of three pooled independent experiments. (**F**) Immunofluorescence staining for CD169 (blue), IgD (magenta), and IgM (yellow) of spleen sections of mice of the indicated genotypes. Scale bars, 100 μm. (**G**) Quantification of average splenic MZ thickness based on immunofluorescence staining. The thickness was measured from the CD169^+^ border to the IgM boundary. The average is representative of three follicles per mouse. Data are representative of two pooled independent experiments. (**H**) Schematic for immunization with heat-inactivated *S.* pneumoniae. I.V., intravenous. (**I**) Total serum levels of anti-phosphorylcholine IgM titers measured by ELISA 5 days postimmunization with *S. pneumoniae*. Mean ± SEM optical density (OD) values for serial dilutions are plotted. Data are representative of one of two independent experiments with similar results. Curves represent the mean response of *n* = 4 mice per genotype. Statistical analyses are done using an unpaired *t* test (A, E, and G), nonparametric test (C), and two-way ANOVA (I). ns, nonsignificant. *P ≤ 0.05, **P ≤ 0.01, ***P ≤ 0.001, and ****P ≤ 0.0001. Each dot represents a biological replicate.

To understand the specific role of Ptpn2 in B cell development and activation, we generated *Ptpn2*^fl/fl^ mb1-*Cre*^+/−^ (*Ptpn2* B cell–specific knockout mice, hereafter referred to as *Ptpn2* BKO mice), in which gene deletion takes place from the pro-B cell stage in the bone marrow onward (*37*). *Ptpn2*^wt/wt^ mb1-*Cre*^+/−^ littermate mice (Ctrl) were used as controls. Ptpn2 deletion was efficient and specific to B cells (fig. S1A). *Ptpn2* BKO mice did not present decreased survival or other signs of morbidity. However, initial characterization of the KO mice revealed a significant increase in spleen size (Fig. 1A), consistent with full-body or hematopoietic-specific *Ptpn2* KO mice (*33*, *35*). High-parameter flow cytometry showed increased absolute numbers of monocytes, CD4^+^ T cells, CD8^+^ T cells, and B cells, reflecting the overall rise in spleen cellularity. However, the relative proportions of major immune cell populations remained unaffected (Fig. 1, B and C, and fig. S1B). Further analysis of *Ptpn2* BKO mice showed no alterations in early B cell development in the bone marrow, except for a small increase in the proportions of pre-B cells (fig. S1C), or in total and recirculating mature B cells in the blood (fig. S1D). Thus, the previously reported developmental block was largely B cell–extrinsic. Notably, we observed a decrease in immature/transitional 1 (T1) B cells in the blood, accompanied by a corresponding increase in T1 B cells in the spleen, with no changes in transitional 2 (T2) B cells (fig. S1, D and E).

A deeper analysis of mature B cells in the spleen showed that the proportions and numbers of MZ B cells were reduced by half in the spleen of *Ptpn2* BKO mice (Fig. 1, D and E). We also observed a slight reduction in Fo B cell proportions but not in the absolute numbers (Fig. 1E), indicating that the decrease in MZ B cells is not a consequence of overall B cell depletion but rather reflects a selective perturbation in this compartment. Consistent with these results, immunofluorescence analysis of spleen sections showed a decrease in the width of the immunoglobulin M–positive (IgM^+^) MZ B cell rim, situated along the outer boundary of the CD169^+^ metallophilic macrophage ring (Fig. 1, F and G). In contrast to control mice, some of the IgM^+^ B cells were instead found in the interfollicular zone in *Ptpn2* BKO mice, a phenotype consistent with MZ B cell activation as they are known to migrate to the center of the follicle in response to certain stimuli to interact with both T cells and dendritic cells (*38*, *39*).

Because of their unique positioning within the marginal sinuses of the spleen, MZ B cells play a critical role in the early control of blood-borne viral infections and encapsulated bacteria by rapidly secreting low-affinity antibodies (*1*, *39*–*41*). To evaluate whether the observed reduction in MZ B cell numbers and redistributed location led to any changes in their function, we injected mice intravenously with heat-inactivated *Streptococcus pneumoniae.* We then measured serum IgM antibodies raised against phosphorylcholine, a component of the bacterial cell wall of this bacterium (Fig. 1H). While *Ptpn2* BKO mice were able to produce anti-phosphorylcholine antibodies, the titers were significantly reduced compared to control mice (Fig. 1I). This reduction is consistent with the diminished, but not completely absent, population of MZ B cells observed in the spleens of *Ptpn2* BKO mice. These findings establish Ptpn2 as a critical mediator of MZ B cell homeostasis, including their frequency, localization, and antibody responses.

### Ptpn2 interacts with and dephosphorylates Lyn to restrict BCR-induced activation

**Fig. 2.**
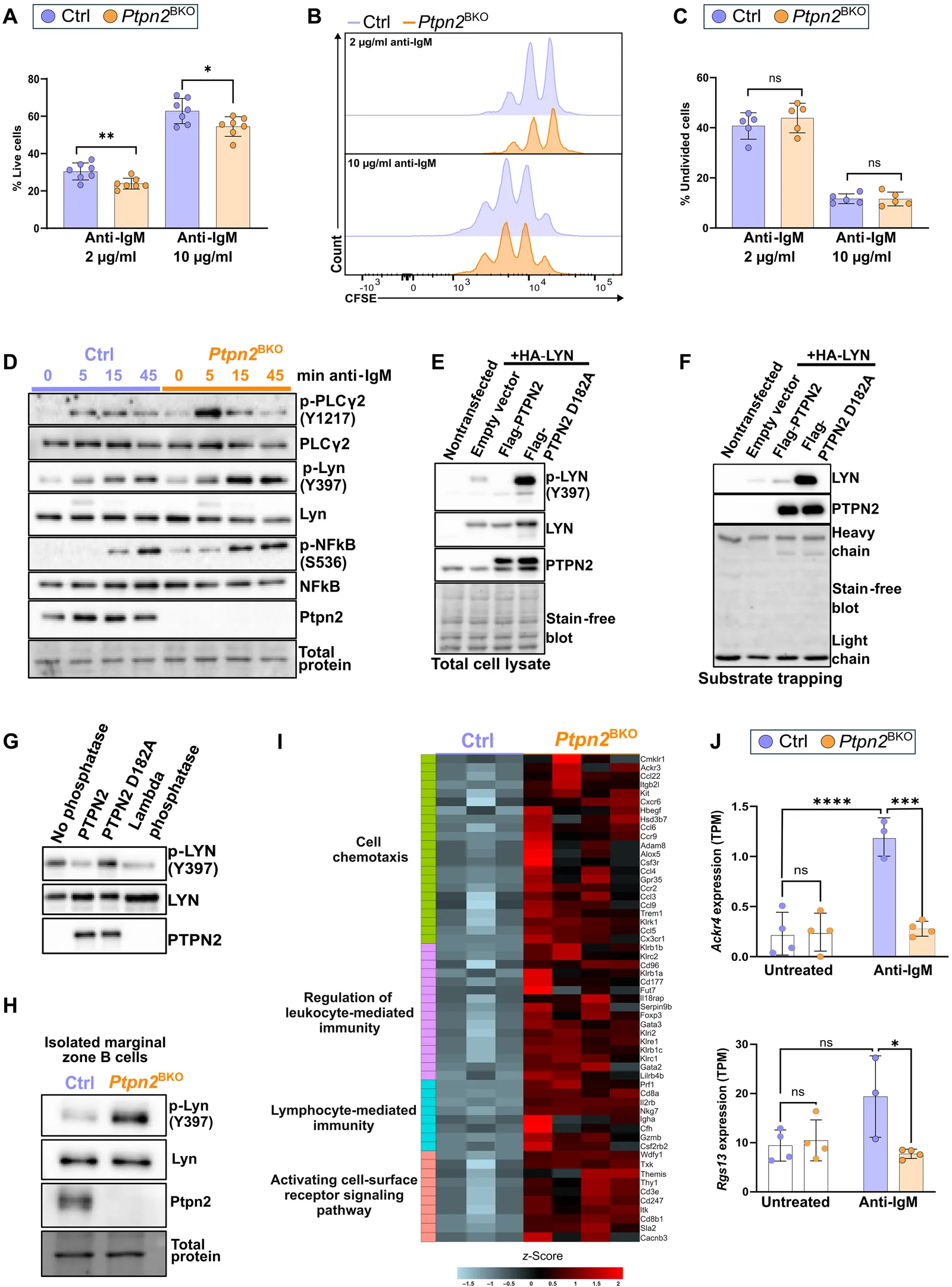
Ptpn2 negatively regulates BCR signaling through direct binding and dephosphorylation of the Lyn kinase. All panels compare *Ptpn2*^fl/fl^ MB1-cre (*Ptpn2*^BKO^) versus *Ptpn2*^wt/wt^MB1-cre (Ctrl) mice. (**A**) Percentages of live B cells after a 72-hour incubation with anti-IgM (2 or 10 μg/ml). Live cells are quantified using a viability dye. Data represent three pooled independent experiments. (**B**) Flow cytometric analysis of CFSE (carboxyfluorescein diacetate succinimidyl ester) proliferation histograms of isolated B cells stimulated ex vivo for 72 hours with anti-IgM (2 or 10 μg/ml). (**C**) Percentages of undivided cells after a 72-hour incubation with anti-IgM (2 or 10 μg/ml). Data represent two pooled independent experiments. (**D**) Immunoblot analysis of phosphorylated (p) and total PLCγ2 Tyr^1217^, Lyn Tyr^397^, and NF-κB Ser^536^ of isolated splenic B cells at the baseline or 5, 15, or 45 min of anti-IgM stimulation. Data are representative of three independent experiments. (**E** and **F**) HEK293T/17 cells were transfected with the indicated constructs followed by immunoblot analysis on (E) total cell lysates and (F) Flag immunoprecipitates. Data are representative of three independent experiments. (**G**) In vitro dephosphorylation assay. Full-length recombinant proteins were co-incubated for 1 hour. p-Lyn was analyzed via immunoblotting. Lambda phosphatase is a positive control for dephosphorylation. Data are representative of three independent experiments. (**H**) Immunoblot analysis of p-Lyn Tyr^397^ in isolated MZ B cells. Data are representative of three independent experiments. (**I**) *z*-Score heatmap analysis for top gene ontology terms performed on significantly up-regulated genes [log_2_FC (log_2_ fold change) > 1, FDR (false discovery rate) < 5%] resulting from bulk RNA-seq analysis on isolated splenic B cells treated ex vivo with anti-IgM for 6 hours. Data are representative of *n* = 4 mice. (**J**) Normalized read counts from RNA-seq of *Ackr4* and *Rgs13*. Statistical analyses are done using an unpaired *t* test (A and C) and two-way ANOVA (J). ns, nonsignificant. *P ≤ 0.05, **P ≤ 0.01, ***P ≤ 0.001, and ****P ≤ 0.0001. Each dot represents a biological replicate.

MZ B cells are susceptible to perturbation in tonic BCR signaling because of their low activation threshold and dependence on survival signals within the splenic niche (*39*, *42*). Consequently, dysregulation of B cell activation pathways frequently results in the loss or altered localization of MZ B cells. For instance, the loss of proteins that negatively regulate BCR signaling has been associated with a reduction or complete absence of MZ B cells in multiple mouse models (*43*–*45*). To investigate the possible role of Ptpn2 in BCR signaling, we isolated total splenic B cells from *Ptpn2* BKO and control mice and stimulated them with anti-IgM F(ab′)_2_ fragments for 72 hours. Although *Ptpn2*-deficient B cells displayed normal activation and proliferation, their survival was significantly reduced in response to both high and low doses of anti-IgM stimulation (Fig. 2, A to C, and fig. S2, A and B). This reduced survival may reflect increased BCR-mediated signaling in the absence of *Ptpn2*, as strong BCR engagement without appropriate secondary signals has been associated with activation-induced cell death (*7*, *46*).

To investigate whether the reduced survival of *Ptpn2*-deficient B cells results from enhanced BCR signaling, we examined the phosphorylation status of key signaling proteins downstream of the BCR. We observed that several proteins involved in this signaling pathway were hyperphosphorylated in *Ptpn2*-deficient B cells compared to controls, namely phospholipase C γ2 (PLCγ2), Lyn, and nuclear factor κB (NF-κB) (Fig. 2D). In particular, the activating residue of the Lyn kinase, Tyr^397^, was found to be hyperphosphorylated. We selected Lyn as the focus of our study as it is the predominant Src-family kinase expressed in B cells and acts upstream of critical effectors such as PLCγ2 and NF-κB, playing a central role in B cell activation and development (*47*–*49*). Although other Src-family kinases have been identified as direct substrates of Ptpn2 in other models such as T cells and fibroblasts (*21*, *22*), it is important to determine whether Lyn is specifically regulated by Ptpn2, given its key role in BCR signaling. Furthermore, mice expressing a constitutively active form of Lyn (Lyn Tyr^507^→Phe) exhibit a complete absence of MZ B cells (*50*), a more severe yet comparable phenotype to the partial reduction observed in Ptpn2 BKO mice.

The Mb1-Cre mouse model of B cell–specific gene deletion is generated by inserting the Cre recombinase into the *mb1* locus, which encodes the CD79a (Igα) protein, thus disrupting the expression of one allele of this gene. Because CD79a is a co-receptor of the BCR, and haploinsufficiency of this gene could influence BCR signaling, we sought to confirm our results in wild-type murine B cells using the Ptpn1/2 competitive inhibitor KQ791. Ptpn2 inhibition increased Lyn Tyr^397^ phosphorylation following BCR stimulation, recapitulating the phenotype observed in *Ptpn2*-deficient B cells (fig. S2C). Given the consistent increase in Lyn Tyr^397^ phosphorylation upon deletion or inhibition of Ptpn2, we next examined whether Lyn is a substrate of this phosphatase. For this purpose, we used the PTPN2 Asp^182^ →Arg (D182A) substrate-trapping mutant, a catalytically inactive variant that retains the ability to bind substrates and form stable complexes, allowing the detection of otherwise transient phosphatase-substrate interactions (*51*, *52*). Coexpression of wild-type PTPN2 with LYN resulted in a marked reduction in its phosphorylation at Tyr^397^, whereas the expression of the D182A mutant led to an increase in phosphorylation at this site (Fig. 2E). In contrast, phosphorylation at the inhibitory site Tyr^507^ of LYN remained unaffected, indicating that PTPN2 selectively targets Tyr^397^ for dephosphorylation (fig. S2D). Immunoprecipitation assays demonstrated that wild-type PTPN2 associates with LYN, and this interaction was enhanced in the presence of the catalytically inactive PTPN2 D182A mutant (Fig. 2F). To determine whether PTPN2 directly dephosphorylates LYN, we conducted an in vitro dephosphorylation assay using recombinant purified active LYN along with wild-type PTPN2 or PTPN2 D182A. PTPN2 directly dephosphorylates LYN at Tyr^397^, whereas this dephosphorylation was abolished in the presence of the D182A mutant (Fig. 2G). To determine whether Lyn Tyr^397^ phosphorylation is specifically elevated in MZ B cells, we analyzed MZ B cells isolated from the spleens of *Ptpn2* BKO mice and observed increased phosphorylation at this site (Fig 2H). These results demonstrate that Lyn is a direct substrate of Ptpn2, and in the absence of this phosphatase, Lyn-driven signaling pathways are hyperactivated in MZ B cells.

To further understand the effect of increased BCR signaling resulting from *Ptpn2* depletion, we first performed RNA sequencing (RNA-seq) on total splenic B cells isolated from the spleens of *Ptpn2* BKO and control mice at the steady state but detected no major transcriptional changes (fig. S2E). We then analyzed gene expression in isolated total splenic B cells after 6 hours of anti-IgM stimulation to evaluate differences under activating conditions. Differential gene expression analysis identified 230 up-regulated and 139 down-regulated genes in *Ptpn2*-deficient B cells (adjusted *P* value cutoff of 0.05 and ≥1 log_2_ fold change). Functional annotation of the up-regulated genes showed an enrichment in genes associated with the gene ontology terms cell chemotaxis, regulation of leukocyte-mediated immunity, lymphocyte-mediated immunity, and activating cell-surface receptor signaling pathway (Fig. 2I), reflecting heightened B cell activation in the absence of *Ptpn2*. These data align with the increased phosphorylation of BCR signaling components previously observed (Fig. 2D). Together, these findings indicate that *Ptpn2* loss enhances B cell activation at both the signaling and transcriptional levels. In addition to analyzing up-regulated genes, we also examined down-regulated transcripts in *Ptpn2*-deficient B cells to explore whether loss of *Ptpn2* also affects the expression of genes that limit B cell activation. Notably, in contrast to control B cells, *Ptpn2*-deficient B cells failed to up-regulate *Ackr4* and *Rgs13* in response to anti-IgM stimulation (Fig. 2J). Ackr4 (atypical chemokine receptor 4) has been shown to limit early plasmablast (PB) differentiation and to prevent activated B cells from accessing the interfollicular zone (*53*). Similarly, RGS13 [regulator of G protein (heterotrimeric guanine nucleotide–binding protein) signaling 13] acts to suppress extrafollicular PB formation and GC expansion (*54*). This indicates that the impaired transcription of regulatory genes may, alongside elevated BCR signaling, drive the premature differentiation and loss of MZ B cells in the absence of *Ptpn2*.

### Ptpn2 limits IFN-γ–induced B cell activation and class-switch recombination

**Fig. 3.**
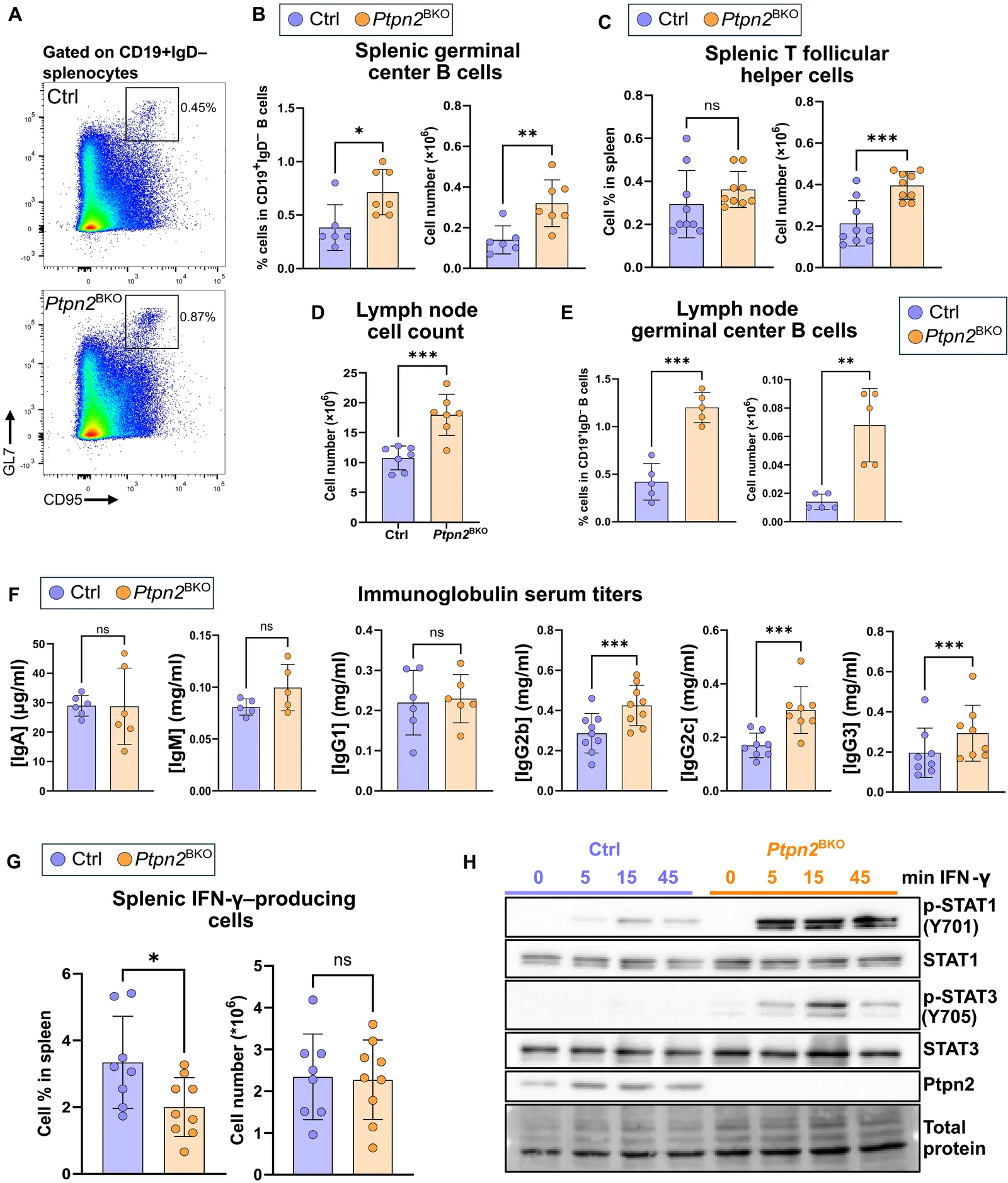
Loss of Ptpn2 in B cells drives spontaneous activation and antibody production in vivo. All panels compare *Ptpn2*^fl/fl^ MB1-cre (*Ptpn2*^BKO^) versus *Ptpn2*^wt/wt^MB1-cre (Ctrl) mice. (**A**) Representative flow cytometry plots of CD19^+^IgD^-^CD95^+^GL7^+^ B cells in the spleen. (**B**) Proportions and absolute cell numbers of splenic GC B cells. Data represent two pooled independent experiments. (**C**) Proportions and absolute cell numbers of splenic T_FH_ cells. Data represent two pooled independent experiments. (**D**) Quantification of lymph node cellularity. Data are representative of pooled counts from two axillary and two inguinal lymph nodes from two independent experiments. (**E**) Frequencies and absolute cell numbers of GC B cells in the lymph nodes. Data represent two pooled independent experiments. (**F**) Serum levels of total IgA, IgM, IgG1, IgG2b, IgG2c, and IgG3 antibody isotypes in naïve mice. Data represent pooled results from at least two independent experiments for each isotype. (**G**) Frequencies and absolute cell numbers of splenic IFN-γ–producing cells in mice of the indicated genotypes. IFN-γ–producing cells are identified via flow cytometry as IFN-γ^+^ cells. Data represent two pooled independent experiments. (**H**) Immunoblot analysis of phosphorylated (p) and total STAT1 Tyr^701^ and STAT3 Tyr^705^ of isolated splenic B cells at the baseline or 5, 15, or 45 min of stimulation with IFN-γ. Data are representative of three independent experiments. Statistical analyses are done using an unpaired t test (B to E and G) and paired *t* test (F). ns, nonsignificant. *P ≤ 0.05, **P ≤ 0.01, and ***P ≤ 0.001. Each dot represents a biological replicate.

While strong BCR signaling can lead to the loss of MZ B cells, it can also promote the activation of Fo B cells (*55*–*57*). Given the enhanced BCR signaling and increased expression of activation-related genes observed in *Ptpn2*-deficient B cells in response to anti-IgM stimulation, we next examined the GC compartment, the activated counterpart of Fo B cells. Consistent with increased Fo B cell activation, we observed a twofold increase in both the frequency and absolute number of spontaneous GC B cells in the spleens of *Ptpn2* BKO mice compared to control mice (Fig. 3, A and B). This expansion of the GC compartment was accompanied by a significant increase in the absolute number of T_FH_ cells in BKO mice (Fig. 3C). Moreover, we detected elevated surface expression of the costimulatory molecules ICOS (inducible T cell costimulator) and CD40L on T_FH_ cells (fig. S3A), indicative of enhanced helper function. Similar to the spleen, lymph nodes from BKO mice exhibited increased cellularity, along with elevated frequencies and total numbers of GC B cells (Fig. 3, D and E), further supporting an enhancement of GC responses in the absence of *Ptpn2*. To assess systemic B cell activation, we measured serum immunoglobulin levels. Among the six major isotypes typically found in mouse serum, IgG2b, IgG2c, and IgG3 were significantly elevated (Fig. 3F). In particular, the production of IgG2c and IgG3 is known to be promoted by IFN-γ, suggesting that *Ptpn2* deficiency may enhance IFN-γ–driven class switching (*15*, *16*). However, the frequency of IFN-γ^+^ cells in the spleen of BKO mice was decreased, while the absolute numbers remained unchanged compared to controls (Fig. 3G), pointing to B cell–intrinsic sensitization to IFN-γ. To study this, we isolated total B cells from the spleens of *Ptpn2* BKO and control mice and stimulated them with IFN-γ. We then analyzed the phosphorylation levels of STAT1 and STAT3, the main mediators of the IFN-γ response in B cells, which are also well-known substrates of Ptpn2 (*58*, *59*). These two TFs were hyperphosphorylated at tyrosine residues in *Ptpn2*-deficient B cells following IFN-γ stimulation, reflecting an intrinsic sensitization of the KO B cells to this cytokine (Fig. 3H). Phosphorylation at the serine sites, Ser^727^ for both STAT1 and STAT3, was also assessed. In contrast to tyrosine phosphorylation, IFN-γ stimulation did not result in an obvious increase in phosphorylation of STAT1 or STAT3 at Ser^727^ under the conditions examined (fig. S3B). This is consistent with a previous study showing that serine phosphorylation of STAT1 and STAT3 in B cells can be regulated independently of tyrosine phosphorylation and may require additional signaling inputs beyond IFN-γ alone (*60*). Moreover, because these residues are serines rather than tyrosines, they are unlikely to be directly regulated by Ptpn2.

To further assess IFN-γ responses in the context of *Ptpn2* depletion, *Ptpn2*-deficient and control B cells were stimulated in vitro for 72 hours with lipopolysaccharide (LPS) and IFN-γ, which led to delayed proliferation and decreased survival, while responses to LPS alone were unaffected (fig. S3, C to E). Delayed cell growth and impaired survival have been associated with sustained STAT1 activation and caspase 3 cleavage (*61*), both of which were evident in *Ptpn2*-deficient B cells in the presence of IFN-γ (fig. S3F). Overall, these data demonstrate that *Ptpn2*-deficient B cells are hypersensitive to IFN-γ and that this might therefore play a role in the increased activation and immunoglobulin secretion we observed in KO mice in vivo.

### *Ptpn2*-deficient B cells exhibit a transcriptional program that favors antibody-secreting cell differentiation

**Fig. 4.**
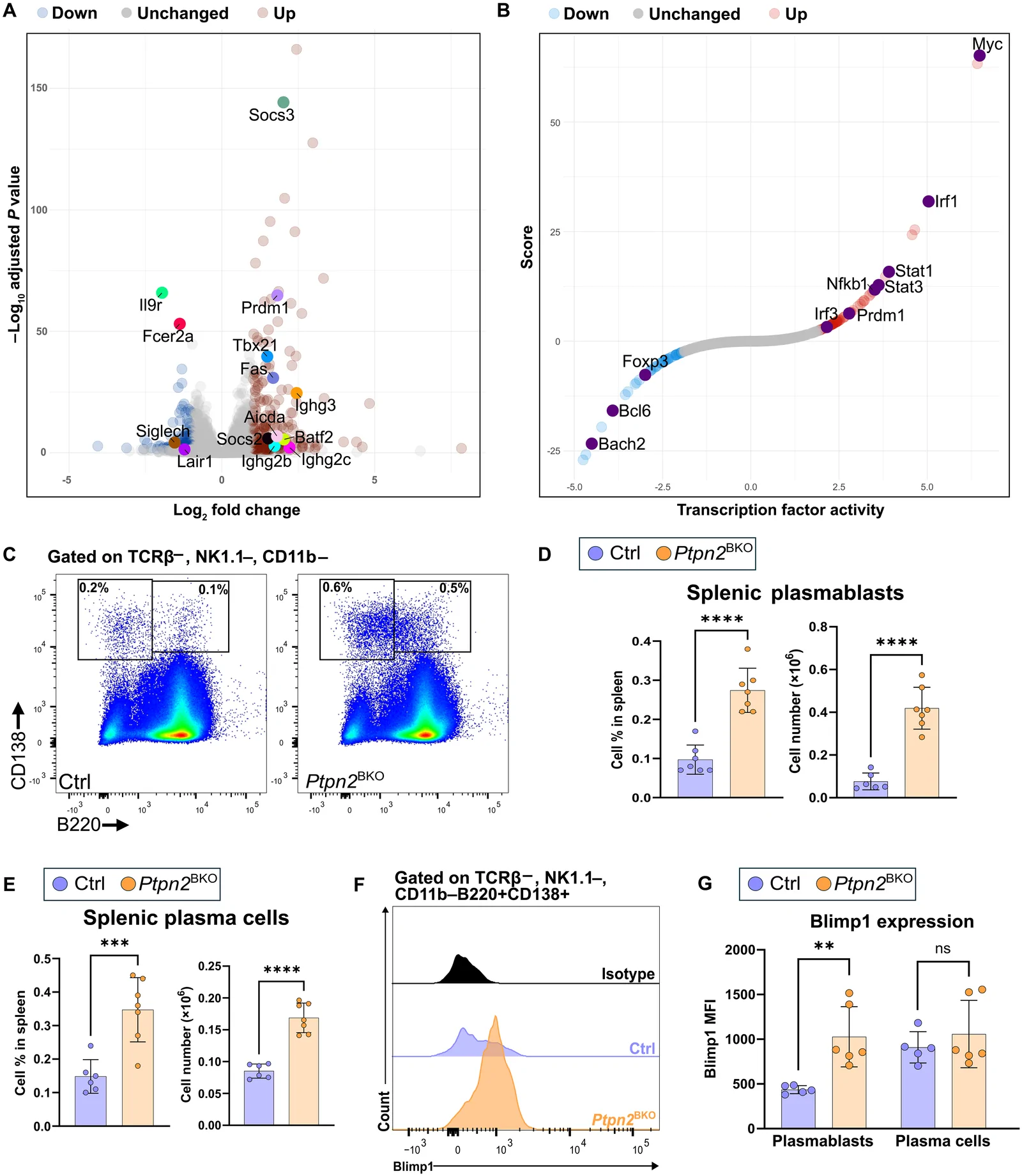
Ptpn2 deletion in B cells promotes IFN-γ–driven PC differentiation. All panels compare *Ptpn2*^fl/fl^ MB1-cre (*Ptpn2*^BKO^) versus *Ptpn2*^wt/wt^MB1-cre (Ctrl) mice. (**A**) RNA-seq volcano plot showing differential gene expression in *Ptpn2* KO versus Ctrl splenic B cells activated ex vivo for 6 hours with IFN-γ. Data are representative of *n* = 4 mice per genotype. (**B**) Plot illustrating TF activity inference for *Ptpn2* KO versus Ctrl splenic B cells activated ex vivo for 6 hours with IFN-γ. Data are representative of *n* = 4 mice per genotype. (**C**) Representative flow cytometry plots of splenic TCRβ^−^NK1.1^−^CD11b^−^B220^lo^CD138^+^ PCs and TCRβ^−^NK1.1^−^CD11b^−^B220^int^CD138^+^ PBs. (**D**) Frequencies and absolute cell numbers of splenic PBs. Data are representative of two pooled independent experiments. (**E**) Frequencies and absolute cell numbers of splenic PCs. Data represent pooled results from two independent experiments. (**F**) Representative flow cytometry histograms of Blimp1 expression in splenic PBs. (**G**) Mean fluorescence intensity (MFI) of Blimp1 in splenic PBs and PCs. Data represent pooled results from two independent experiments. Statistical analyses for all figures are done using an unpaired *t* test. ns, nonsignificant. **P ≤ 0.01, ***P ≤ 0.001, and ****P ≤ 0.0001. Each dot represents a biological replicate.

STAT1 and STAT3 regulate gene expression programs that drive B cell activation and differentiation into antibody-secreting cells in response to cytokine signaling (*62*–*66*). Given the critical role of Ptpn2 in regulating STAT1/3 activity, we performed RNA-seq on total splenic B cells isolated from control and *Ptpn2* BKO mice following a 6-hour stimulation with IFN-γ. In total, we identified 348 differentially expressed genes between both groups (Fig. 4A). Among other genes, we saw an increase in *Aicda, Fas*, and *Batf2* expression in KO cells, which are required for B cell activation and class-switch recombination (*67*–*69*). This was accompanied by elevated levels of immunoglobulin transcripts *Ighg2b*, *Ighg2c*, and *Ighg3*, consistent with the increased serum titers of these isotypes observed in naïve *Ptpn2* BKO mice (Fig. 3F). Expression of the TFs Prdm1 (*Blimp1*) and Tbx21 (*T-bet*) was markedly up-regulated in KO B cells. These genes are important drivers of PC differentiation, suggesting enhanced progression toward the antibody-secreting phenotype (*14*, *70*, *71*). Notably, the absence of *Ptpn2* not only enhances the expression of genes associated with terminal B cell differentiation but also leads to the down-regulation of genes that may normally restrain B cell activation. In particular, the expression of *Fcer2a* (CD23) and *Lair1* (LAIR-1) (*72*, *73*), two negative regulators of BCR signaling, was significantly reduced in *Ptpn2*-deficient B cells. To identify TFs that may be driving the altered gene expression program in *Ptpn2*-deficient B cells, we performed TF activity analysis using CollecTRI (Fig. 4B) (*74*). Consistent with our previous Western blot results (Fig. 3H), this analysis identified STAT1 and STAT3 among the top TFs with increased activity in the absence of Ptpn2. Several interferon-related TFs, including IRF1, IRF3, and NF-κB, also showed enhanced activity (Fig. 4B). Notably, in addition to being up-regulated at the transcript level, Blimp1 also exhibited increased inferred activity. This analysis also revealed that the activity of Bcl6 and Bach2, two TFs that limit PC differentiation (*75*, *76*), was decreased in KO B cells (Fig. 4B).

Together, our RNA-seq data point to increased differentiation of B cells into antibody-secreting cells in response to IFN-γ. To further study how the increased transcription of genes favoring antibody-secreting cell differentiation might translate to observations in vivo, we then focused on splenic PBs and PCs, which are largely recently generated compared to PCs in the bone marrow. We found that the frequencies and absolute numbers of both populations of antibody-secreting cells were increased in KO mice compared to controls (Fig. 4, C to E). These findings were observed in unimmunized mice, indicating that *Ptpn2* deficiency predisposes B cells to spontaneous differentiation into PBs and PCs, independent of exogenous antigen stimulation. During differentiation, the expression of Blimp1 increases as B cells progress from PBs to PCs (*77*). We observed that the levels of Blimp1 expression in *Ptpn2*-deficient PBs were significantly higher compared to controls, and the expression levels were comparable to that of PCs (Fig. 4, F and G), possibly contributing to the increase in the frequency of antibody-secreting cells. Together, our data demonstrate that Ptpn2 deficiency leads to transcriptional reprogramming that favors the differentiation of B cells into antibody-secreting cells in response to IFN-γ.

### Loss of *Ptpn2* leads to IFN-γ–driven antibody responses upon immunization

**Fig. 5.**
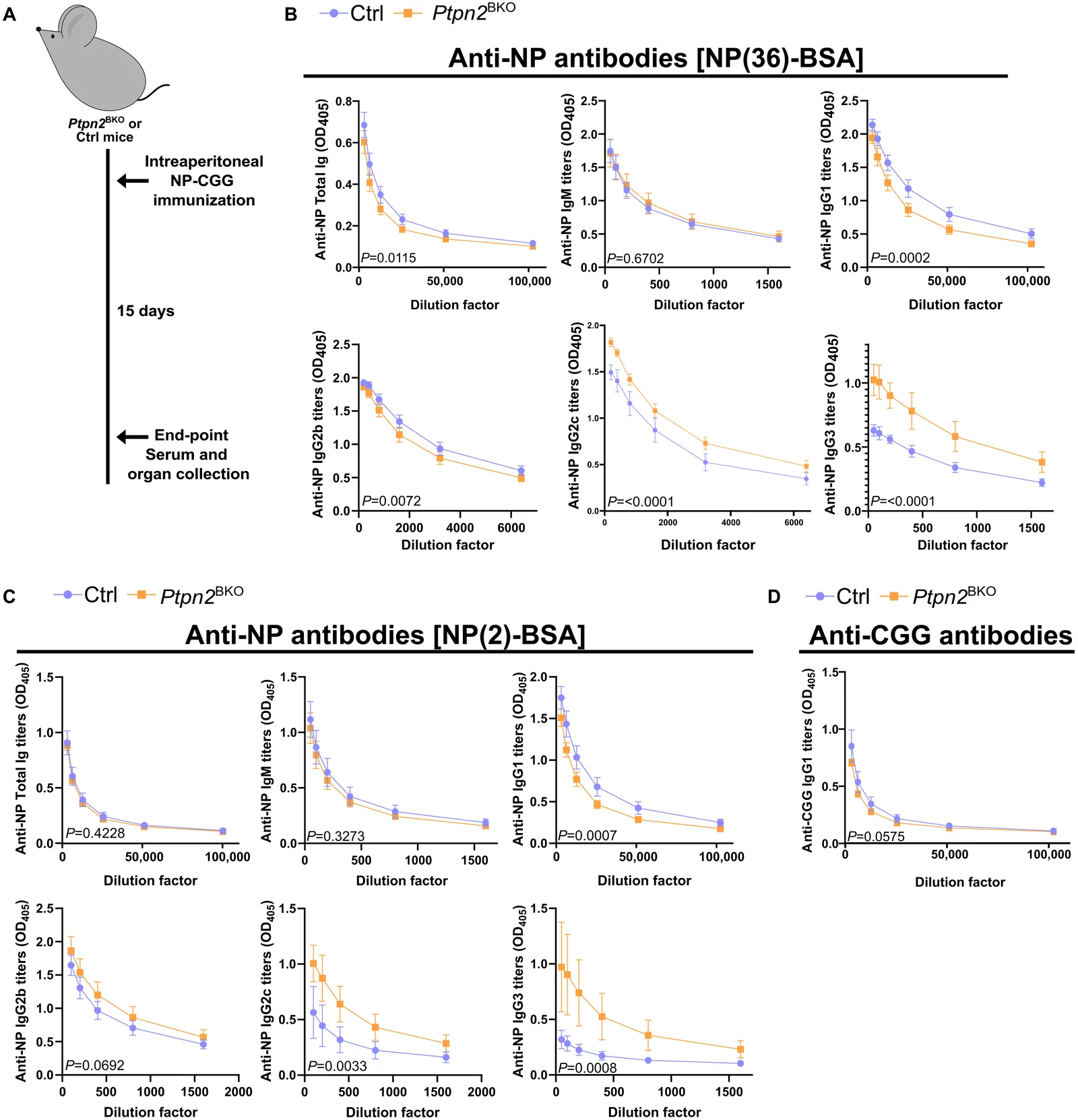
Altered antibody secretion profile in Ptpn2-deficient B cells following immunization with a protein antigen. All panels compare *Ptpn2*^fl/fl^ MB1-cre (*Ptpn2*^BKO^) versus *Ptpn2*^wt/wt^MB1-cre (Ctrl) mice. (**A**) Schematic for immunization with NP-CGG emulsified in alum (primary response). (**B**) Serum levels of anti-NP total Ig, IgM, IgG1, IgG2b, IgG2c, and IgG3 titers measured by ELISA 14 days postimmunization with NP-CGG. Mean ± SEM OD values for serial dilutions are plotted. Data are representative of one of two independent experiments with *n* = 4 mice per genotype each. Curves represent the mean response of *n* = 4 mice per genotype. (**C**) Serum titers of anti-NP high-affinity total Ig, IgM, IgG1, IgG2b, IgG2c, and IgG3 titers measured by ELISA 14 days postimmunization with NP-CGG. Mean ± SEM OD values for serial dilutions are plotted. Data are representative of one of two independent experiments with *n* = 4 mice per genotype each. Curves represent the mean response of *n* = 4 mice per genotype. (**D**) Serum levels of anti-CGG IgG1 titers measured by ELISA 14 days postimmunization with NP-CGG. Mean ± SEM OD values for serial dilutions are plotted. Data are representative of one out of two independent experiments with *n* = 4 mice per genotype each. Curves represent the mean response of *n* = 4 mice per genotype. Statistical analyses for all figures are done using a two-way ANOVA.

At steady state, *Ptpn2* BKO mice exhibited elevated serum levels of IgG2c and IgG3 isotypes, which are typically associated with IFN-γ–driven type 1 (Th1) responses. Consistently, *Ptpn2*-deficient B cells showed increased transcription of these same isotypes in response to IFN-γ stimulation. On the basis of these findings, we hypothesized that *Ptpn2* deficiency would skew the antibody response toward IFN-γ–associated isotypes following immunization. To examine the role of *Ptpn2* in PC differentiation and antigen-specific antibody responses, we immunized mice with the T-dependent antigen NP-CGG in alum (Fig. 5A). This model typically elicits a type 2 (Th2)–skewed response and leads to the preferential production of IgG1 (*78*), providing a useful context to detect shifts toward Th1-associated isotypes. Fourteen days postimmunization, *Ptpn2* BKO mice exhibited a modest reduction in total anti-nitrophenol (anti-NP) antibody titers, particularly in the IgG1 and IgG2b isotypes, compared to controls (Fig. 5B). In contrast, levels of anti-NP IgG2c and IgG3 were significantly elevated, while anti-NP IgM titers remained unchanged. Similar isotype skewing was observed in the high-affinity anti-NP antibody response, although overall high-affinity titers were not significantly affected (Fig. 5C). Anti–chicken gamma globulin (anti-CGG) IgG1, the only detectable anti-CGG isotype, was also unaffected (Fig. 5D). Despite these changes in antibody profiles, the frequency of splenic GC B cells was comparable between groups (fig. S4A). However, *Ptpn2* BKO mice displayed increased proportions and absolute numbers of PBs without a corresponding increase in PCs (fig. S4, B and C).

**Fig. 6.**
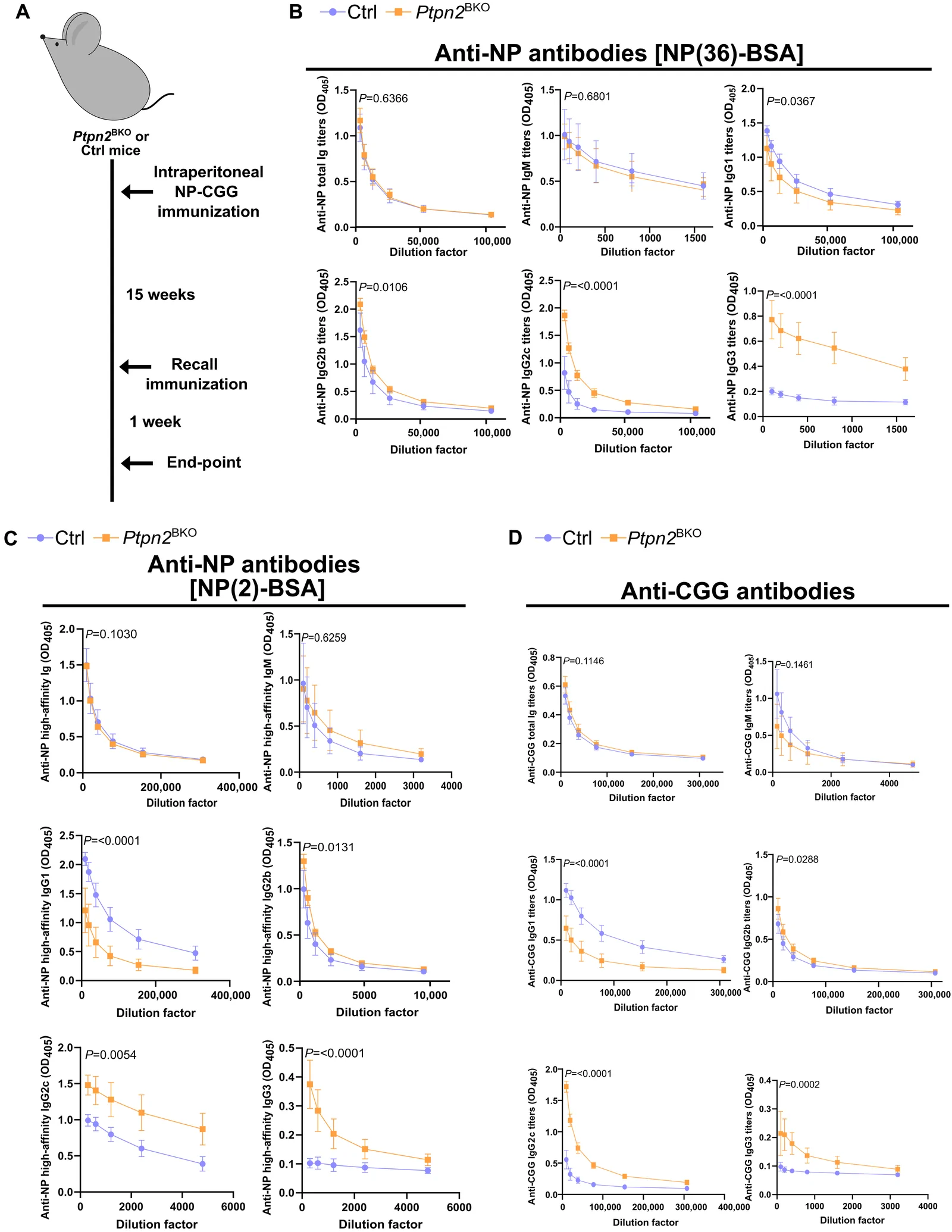
Ptpn2 restrains type I secondary B cell antibody responses. All panels compare *Ptpn2*^fl/fl^ MB1-cre (*Ptpn2*^BKO^) versus *Ptpn2*^wt/wt^MB1-cre (Ctrl) mice. (**A**) Schematic for primary and recall immunization with NP-CGG emulsified in alum. (**B**) Serum titers of anti-NP total Ig, IgM, IgG1, IgG2b, IgG2c, and IgG3 titers measured by ELISA 7 days post–recall immunization. Mean ± SEM OD values for serial dilutions are plotted. Curves represent all mice analyzed (*n* = 5 per genotype). (**C**) Serum levels of anti-NP high-affinity total Ig, IgM, IgG1, IgG2b, IgG2c, and IgG3 titers measured by ELISA 7 days post–recall immunization. Mean ± SEM OD values for serial dilutions are plotted. Curves represent the mean response of *n* = 5 mice per genotype. (**D**) Serum levels of anti-CGG total Ig, IgM, IgG1, IgG2b, IgG2c, and IgG3 titers measured by ELISA 7 days post–recall immunization. Mean ± SEM OD values for serial dilutions are plotted. Curves represent the mean response of *n* = 5 mice per genotype. Statistical analyses for all figures are done using a two-way ANOVA.

To assess whether these alterations persisted in secondary B cell responses, we performed a recall immunization 15 weeks after the primary challenge (Fig. 6A). Following the secondary immunization, total anti-NP antibody titers were comparable between *Ptpn2* BKO and control mice, with a slight increase in anti-NP IgG2b in KO animals (Fig. 6B). Notably, anti-NP IgG1 remained reduced, while IgG2c and IgG3 were markedly elevated in *Ptpn2*-deficient mice, mirroring the primary response (Fig. 6B). This isotype distribution was also observed in high-affinity NP and CGG recall antibody responses (Fig. 6, C and D). Last, an increase in PB frequency was also observed in response to the recall immunization, with a concomitant increase in PC absolute numbers (fig. S4, E and F). Notably, class-switch recombination to IgG1 was not affected in vitro in the absence of exogenous IFN-γ (fig. S4G). These findings demonstrate that *Ptpn2* BKO mice present a shift in immunoglobulin isotype distribution in both primary and secondary responses. In particular, they preferentially produce isotypes generated in response to IFN-γ, consistent with the heightened response to this cytokine in the absence of *Ptpn2*. Moreover, these results further support the proposed function of this phosphatase in negatively regulating the terminal differentiation of antibody-secreting cells.

### *Ptpn2* BKO mice present improved humoral immunity in response to influenza A virus infection

**Fig. 7.**
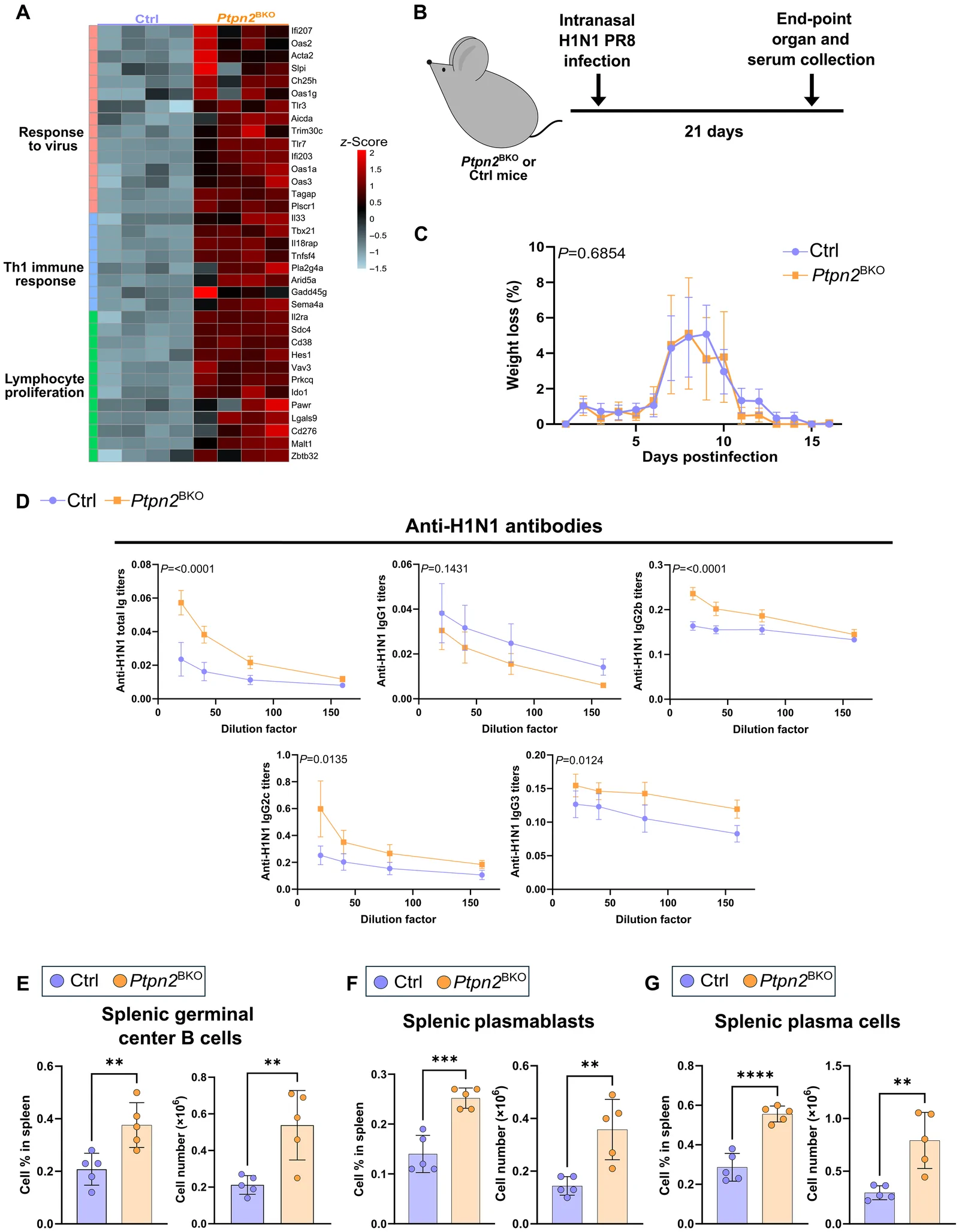
Ptpn2 deletion in B cells increases antibody responses against influenza H1N1. All panels compare *Ptpn2*^fl/fl^MB1-cre (*Ptpn2*^BKO^) versus *Ptpn2*^wt/wt^MB1-cre (Ctrl) mice. (**A**) *z*-Score heatmap showing enrichment of top gene ontology terms derived from significantly up-regulated genes (log_2_FC > 1, FDR < 5%) resulting from bulk RNA-seq of isolated splenic B cells treated ex vivo with IFN-γ for 6 hours. Data depict *n* = 4 mice per genotype. (**B**) Schematic for intranasal infection with influenza A virus H1N1 PR8 (primary infection). (**C**) Percent body weight loss at the indicated times post–H1N1 infection. Data are representative of one of two independent experiments with *n* = 4 or 5 mice per group each. (**D**) Serum levels of anti-H1N1 total Ig, IgG1, IgG2b, IgG2c, and IgG3 titers measured by ELISA 21 days after intranasal infection with 30 PFU/20 g of body weight of H1N1 PR8. Mean ± SEM OD values for serial dilutions are plotted. Data are representative of one of two independent experiments with *n* = 4 or 5 mice each. Curves represent the mean response of *n* = 4 or 5 mice per genotype. (**E** to **G**) Frequencies and absolute cell numbers of splenic (E) GC B cells, (F) PBs, and (G) PCs in mice of the indicated genotypes 21 days post–H1N1 infection. Statistical analyses are done using a two-way ANOVA (C and D) or unpaired *t* test (E to G). ns, nonsignificant. **P ≤ 0.01, ***P ≤ 0.001, and ****P ≤ 0.0001. Each dot represents a biological replicate.

We next wanted to understand the biological implications of the absence of *Ptpn2* on the increased IFN-γ response and changes in antibody isotype distribution observed following immunization. Functional enrichment analysis of differentially expressed genes in IFN-γ–treated B cells from our RNA-seq data (Fig. 4A) revealed an enhanced expression of genes associated with antiviral responses, Th1 immune response, and lymphocyte proliferation (Fig. 7A). These findings align with our immunization data, as IFN-γ–driven production of IgG2c and IgG3 is a hallmark of Th1 immune responses. Th1 responses are often raised in the response against intracellular pathogens, such as viruses (*79*, *80*). On the basis of these observations, we hypothesized that *Ptpn2* BKO mice would exhibit enhanced antiviral humoral immunity, characterized by increased titers of virus-specific antibodies. To test this, we infected naïve *Ptpn2* BKO and control mice intranasally with 30 plaque-forming units (PFU) per 20 g of body weight of influenza A (H1N1) virus (strain A/Puerto Rico/8/34) (Fig. 7B). Weight loss, which is proportional to disease severity, was comparable between groups (Fig. 7C), suggesting that *Ptpn2* deficiency does not exacerbate clinical disease despite altering immune dynamics. By day 21 postinfection, *Ptpn2* BKO mice exhibited significantly increased serum levels of flu-specific total Ig, IgG2b, IgG2c, and IgG3, whereas IgG1 levels were reduced (Fig. 7D). These humoral responses were accompanied by elevated proportions and absolute numbers of splenic GC B cells, PBs, and PCs (Fig. 7, E to G). This observation supports the idea that the transcriptional priming observed ex vivo translates into an increased effector B cell response during active infection. Together, these data demonstrate that B cell–specific *Ptpn2* deficiency enhances the magnitude of the antibody response to primary influenza infection, particularly through elevated titers of anti-H1N1 IgG2c, which have been associated with protective responses in previous studies (*81*, *82*).

**Fig. 8.**
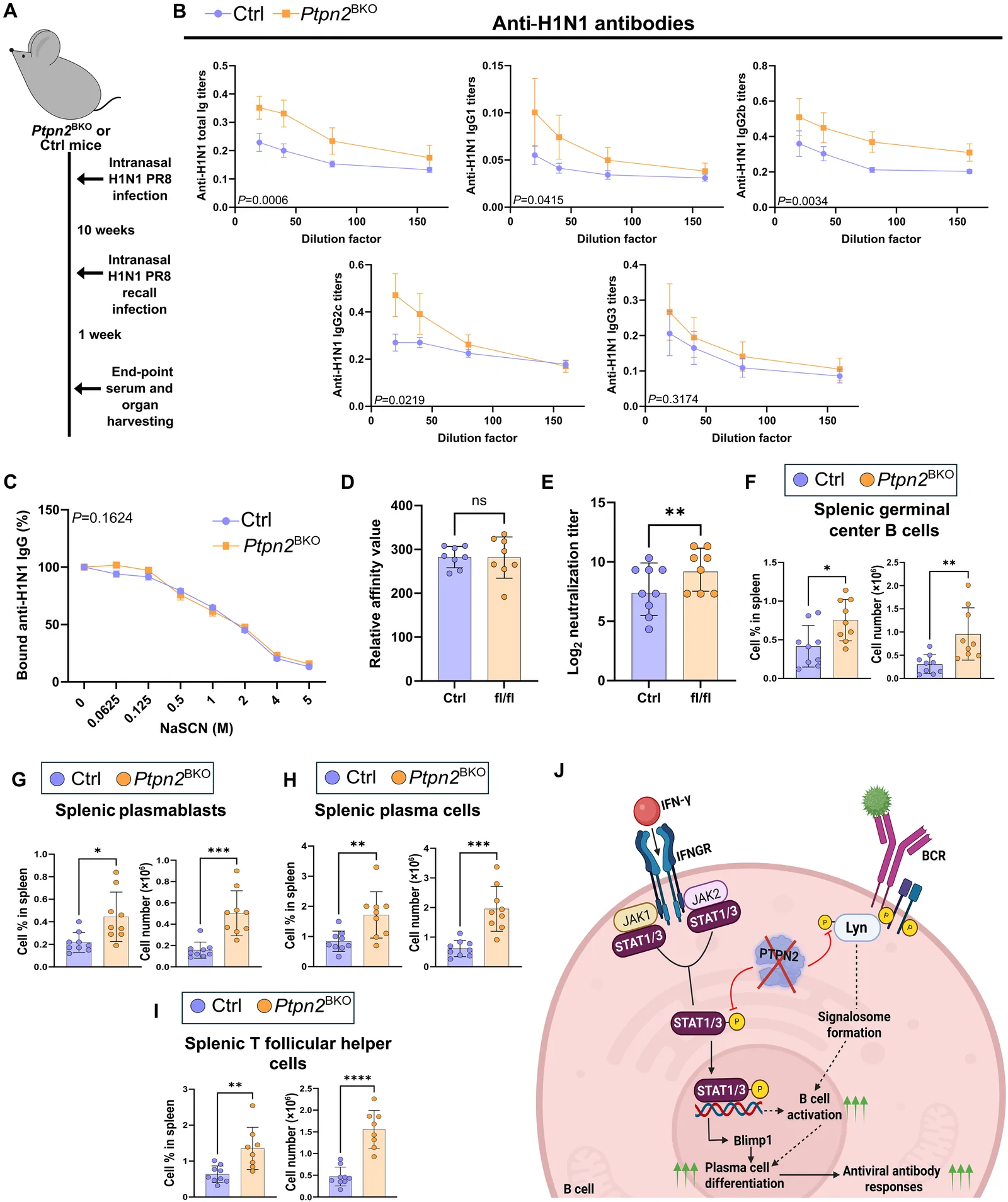
Ptpn2 deficiency enhances recall humoral responses to influenza H1N1 reinfection. All panels compare *Ptpn2*^fl/fl^MB1-cre (*Ptpn2*^BKO^) versus *Ptpn2*^wt/wt^MB1-cre (Ctrl) mice. (**A**) Schematic for intranasal infection with influenza A virus H1N1 PR8 (primary and recall infection). (**B**) Serum titers of anti-H1N1 total Ig, IgG1, IgG2b, IgG2c, and IgG3 measured by ELISA 7 days after recall infection with 60 PFU/20 g of body weight of H1N1. Mean ± SEM OD values for serial dilutions are plotted. Data represent one of two independent experiments with *n* = 4 or 5 mice each. (**C**) H1N1-specific IgG avidity was assessed by NaSCN displacement ELISA. The fraction of antibody remaining bound after each NaSCN wash was calculated as the absorbance relative to untreated control wells. Mean ± SEM OD values are shown for each NaSCN concentration. Data are representative of two pooled experiments with *n* = 4 mice per genotype each. (**D**) Calculated relative affinity values (RAV) for the IgG secondary response to H1N1 infection. Data represent two pooled independent experiments with *n* = 4 mice per genotype per experiment. (**E**) Determination of neutralizing anti-H1N1 antibody titers using a microneutralization assay. Neutralizing titers were defined as the highest serum dilution that prevented the virus-induced cytopathic effect. Titers were log_2_-transformed for statistical analysis. Data represent pooled results from two independent experiments. (**F** to **I**) Frequencies and absolute numbers of splenic (F) GC B cells, (G) PBs, (H) PCs, and (I) T_FH_ cells 7 days post–secondary H1N1 infection. Data represent pooled results from two independent experiments. (**J**) Model describing the role of Ptpn2 in B cell activation and antiviral immunity. Created in BioRender. J.-F. Theberge (2026), https://biorender.com/f86rnmy. Statistical analyses are done using a two-way ANOVA (B and C), unpaired *t* test (D and F to I), or paired *t* test (E). ns, nonsignificant. *P ≤ 0.05, **P ≤ 0.01, ***P ≤ 0.001, and ****P ≤ 0.0001. Each dot represents a biological replicate.

To determine whether this enhanced immunity extended to secondary responses, we reinfected mice 10 weeks post–primary infection with a dose of 60 PFU/20 g of H1N1 (Fig. 8A). While we had previously observed this dose to be lethal in naïve mice, it is sublethal upon rechallenge, as confirmed by the survival of all mice in both the control and *Ptpn2* BKO groups. This sublethal model enables detection of subtle immune differences such as shifts in antibody isotypes or GC responses, which would be masked by the severe inflammation of lethal infection. KO mice presented significantly elevated anti-H1N1 antibody titers across most isotypes, with IgG3 showing a nonsignificant increase (Fig. 8B). Affinity maturation of IgG, assessed via sodium thiocyanate (NaSCN) displacement enzyme-linked immunosorbent assay (ELISA), was similar between groups (Fig. 8, C and D). Anti-H1N1 antibodies in the sera of *Ptpn2* BKO mice exhibited enhanced neutralization capacity in an in vitro microneutralization assay (Fig. 8E). Flow cytometry revealed increased frequencies and numbers of GC B cells, PBs, PCs and T_FH_ cells in the spleens of *Ptpn2* BKO mice (Fig. 8, F to I). Moreover, the proportions and absolute numbers of PBs and PCs were increased in the mediastinal lymph node without changes in GC B cells (fig. S5, A to C), consistent with enhanced local humoral responses in addition to systemic responses. These findings indicate that Ptpn2 functions to restrain Th1-associated humoral responses and that its absence enhances both primary and recall B cell responses associated with protective immunity. Overall, we have demonstrated that *Ptpn2* deficiency enhances antiviral B cell immunity by promoting differentiation into IgG2c-secreting cells, thereby increasing the production of virus-specific antibodies.

## DISCUSSION

The differentiation of B cells into PCs is a tightly regulated process, critical for the generation of protective antibody responses. Understanding the molecular pathways that drive this differentiation is central to improving vaccine effectiveness and modulating immune responses in acute and chronic infections. A key factor in determining the effectiveness of the antibody response is the type of immunity that is engaged in response to infection, namely Th1 or Th2. Each type of immunity is characterized by a specific group of cytokines, which influence class-switch recombination and ultimately determine the quality of the humoral response to an infection. Herein, we have uncovered a previously unknown role for Ptpn2 in negatively regulating PC differentiation. Mechanistically, Ptpn2 directly dephosphorylates Lyn and STAT1/3, thus attenuating BCR and IFN-γ signaling, respectively. Loss of *Ptpn2* in B cells leads to hyperresponsiveness to IFN-γ, promoting Th1-skewed antibody responses, as well as enhanced differentiation into antibody-secreting cells following influenza virus infection (Fig. 8J).

In this study, we present an in-depth characterization of a B cell–specific *Ptpn2* KO mouse model. Notably, in contrast to previously published full-body and hematopoietic-specific *Ptpn2* KO models, we observed no defects in B cell maturation within the bone marrow (*33*–*35*). This finding suggests that the blockages in B cell development reported in broader KO models are likely due to extrinsic effects from other hematopoietic or stromal compartments. Despite the absence of developmental abnormalities or overt morbidity in our mouse model, several key immunological phenotypes were recapitulated. For instance, we observed increased spleen and lymph node cellularity, increased frequencies of GC B cells, and higher serum levels of class-switched immunoglobulins. Consistent with our observations, a recent study using a B cell–specific *Ptpn2* KO mouse model similarly reported no overt signs of disease or autoimmunity. However, more detailed analyses revealed evidence of B cell dysregulation, including increased immune cell infiltration in peripheral tissues and the gradual emergence of autoantibodies, as well as expansion of pro-inflammatory B cell populations over time (*36*). Together, these findings and our study support a model in which B cell–intrinsic loss of *Ptpn2* lowers the threshold for activation, predisposing B cells to heightened responses that may manifest differently depending on the immunological context.

The enhanced B cell activation resulting from *Ptpn2* deletion observed in our model is likely supported, at least in part, by elevated T_FH_ cell frequencies with increased expression of costimulatory molecules. However, how B cell–specific *Ptpn2* deficiency affects the cross-talk between GC B cells and T_FH_ cells, including cytokine signals and costimulatory interactions, remains unclear and should be addressed in future studies. Nevertheless, while some questions remain regarding the role of *Ptpn2* in overall adaptive immunity, the elevated lymphocyte activation observed in *Ptpn2* BKO mice aligns well with clinical features observed in patients with autoimmune disease who carry *PTPN2* loss-of-function mutations or variants that lead to decreased PTPN2 expression (*32*). Notably, while the Th1-skewed humoral profile in *Ptpn2* BKO mice is advantageous in the context of intracellular pathogens such as influenza, this same bias may exacerbate immunopathology or autoimmunity in other settings. Through this work, we have begun to dissect the relative contribution of *Ptpn2* deficiency in B cells to the broader immunological symptoms observed in patients. Knowing how Ptpn2 functions in specific immune compartments is important for the development of therapies targeting this phosphatase in autoimmune or inflammatory conditions.

In addition to the observed increase in B cell activation, loss of *Ptpn2* in B cells resulted in a notable reduction in the frequency of MZ B cells. The decrease in this population may reflect a disruption of MZ B cell homeostasis and localization within the spleen. Altered positioning of MZ B cells has been previously linked to increased self-antigen presentation within the follicle, leading to enhanced GC responses (*83*). This mechanism may partially explain the elevated GC responses observed in our model. Such alterations in MZ B cell homeostasis are consistent with increased basal BCR signaling in the absence of Ptpn2. We identified Lyn, an Src family kinase involved in early BCR signaling, as a direct substrate of Ptpn2. While other Src family kinases have also been reported as substrates of Ptpn2, our findings highlight a previously unrecognized role for this phosphatase in modulating proximal BCR signaling (*21*, *22*). Enhanced BCR signaling has been previously associated with impaired MZ B cell survival, as well as premature activation and differentiation (*43*–*45*). The increased serum IgG3 titers, an isotype linked to extrafollicular responses, suggest that MZ B cells may spontaneously differentiate into PBs at the expense of their maintenance in the MZ (*84*, *85*). While enhanced PC differentiation may be desirable in the context of viral infections, it can also have detrimental effects depending on the nature of the pathogen. In our model, the premature differentiation of MZ B cells compromises the response to blood-borne bacteria. Some patients carrying *PTPN2* variants have also been shown to present recurrent infections or primary immunodeficiencies (*86*). These findings raise the possibility that *PTPN2* mutations contribute to the immunodeficiency observed in some patients by disrupting B cell–mediated antibacterial responses, although further studies are needed to confirm this link.

BCR signaling strength has likewise been shown to be a key determinant of terminal B cell differentiation, with high-affinity signals promoting active selection into the PC compartment (*87*, *88*). Along with limiting antigen receptor signaling, Ptpn2 is also known to modulate cytokine responses in immune cells, which also govern B cell differentiation. The differentiation of B cells into antibody-secreting cells is governed by the interplay of cytokine signals and transcriptional regulators, such as the IFN-γ-JAK-STAT signaling axis (*59*, *65*). The roles of STAT1/3 in promoting B cell activation and class-switch recombination are well documented, with STAT3 playing a prominent role in promoting antibody-secreting cell differentiation (*63*–*65*). By attenuating STAT1/3 activity, Ptpn2 indirectly regulates a transcriptional network that includes up-regulation of Blimp1 and suppression of Bcl6 and Bach2, two well-established antagonists of PC fate. This research positions Ptpn2 as a critical regulator that integrates cytokine and antigen receptor signals to restrain PC differentiation and control humoral immunity.

Beyond its role in regulating PC differentiation, Ptpn2 also appears to influence the quality of antibody responses. Despite the use of NP-CGG with alum, which is a classical Th2-polarizing immunogen (*78*), we observed marked skewing toward Th1 immunity in KO mice. This shift was observed across multiple immunization models and influenza virus challenge and aligns with enhanced IFN-γ responsiveness in *Ptpn2*-deficient B cells. Th1 antibody responses are essential for protection against intracellular pathogens such as SARS-CoV-2 (severe acute respiratory syndrome coronavirus 2), influenza virus, and *Mycobacterium tuberculosis* and are a desirable outcome for vaccines targeting these organisms (*81*, *89*–*91*). However, the long-term efficacy of vaccines against such pathogens remains suboptimal, partly due to the decline of antigen-specific PCs over time (*92*, *93*). Notably, *Ptpn2*-deficient mice mounted a more robust recall B cell response to influenza infection, consistent with their Th1-biased humoral profile. While the relative contribution of increased primary humoral responses versus enhanced memory B cell sensitivity to this phenotype remains to be fully defined and thus represents a limitation of the current study, our work nonetheless supports a role for Ptpn2 in restraining humoral antiviral immunity. These findings suggest that pharmacologically targeting Ptpn2 could enhance B cell–driven protective immunity against infectious diseases. Such strategies may be particularly beneficial in patients with poor IFN-γ responses or suboptimal vaccine-induced humoral immunity. Ptpn2 inhibitors have gained notoriety in recent years because of their potential to enhance antitumor immunity (*27*, *94*, *95*). However, currently available Ptpn2 inhibitors also target the closely related phosphatase Ptpn1 because of the high degree of similarity of their catalytic sites (*96*, *97*). While dual inhibition of Ptpn1 and Ptpn2 may further enhance immune responses to infectious diseases, the impact of simultaneously targeting these phosphatases in B cells remains poorly defined. Until these effects are better studied, the development of specific Ptpn2 inhibitors remains a critical step toward the improvement of humoral immunity and vaccine efficacy.

Together, our study provides a comprehensive characterization of the intrinsic role of Ptpn2 in B cell development, activation, and immune responses. We have uncovered a general role for this enzyme in limiting the differentiation of B cells into antibody-secreting cells by modulating transcriptional responses to extracellular signals. With this work, we provide insight into the B cell–intrinsic contribution to the established association between PTPN2 and autoimmune disease. Last, we also bring forward PTPN2 as a previously unrecognizedtarget for the improvement of B cell responses to vaccines against infectious diseases.

## MATERIALS AND METHODS

### Study design

The role of Ptpn2 in B cell development and activation was assessed using a *Ptpn2* B cell–specific KO mouse model. We performed a comprehensive characterization of immune cell populations and signaling pathways using flow cytometry, ELISA, Western blotting, immunoprecipitation, and RNA-seq. We also studied the effects of *Ptpn2* deficiency in B cell primary and recall responses using two different models: immunization with a protein antigen and infection with influenza A virus. For animal experiments, age- and sex-matched male and female mice were used, and the investigators were not blinded to the genotypes.

### Mice

*Ptpn2*^fl/fl^ mice on a C57BL/6 background were generated in our laboratory, as previously described (*98*). Mice with specific *Ptpn2* deletion in B cells were generated by crossing *Ptpn2*^fl/fl^ mice with mb1-*cre* mice purchased from the Jackson Laboratory (strain no. 020505). Control mice for experiments were *Ptpn2*^wt/wt^ Cre^+/−^ littermates. All animal experiments were performed on mice 8 to 12 weeks of age, unless otherwise specified. Both male and female mice were used in all experiments. Experimental groups were age and sex matched across genotypes. All procedures were done according to the Canadian Council on Animal Care ethical regulations and were approved by the McGill University Research and Ethics Animal committee (Animal Use Protocol MCGL-3188).

### Flow cytometry

Single-cell suspensions were obtained by crushing mouse spleens with a syringe plunger on a 70-μm filter. Cell suspensions were subsequently treated with Lympholyte-M (cat. no. CL5030, Cedarlane) for red blood cell removal following the manufacturer’s instructions. Blood was collected and immediately treated with BD Pharm Lyse Lysing Buffer (cat. no. 555899, BD Biosciences) for red blood cell removal following the manufacturer’s instructions. For cell surface staining, single-cell suspensions obtained from mouse organs or blood were treated with anti-CD16/32 (cat. no. 14-0161-81, eBioscience) at room temperature for 5 min followed by incubation with combinations of the antibodies listed in table S1 at 4°C for 30 min. For intracellular staining, cells were fixed and permeabilized with FoxP3/Transcription Factor Fixation/Permeabilization Concentrate and Diluent (cat. no. 00-5521-00, eBioscience) according to the manufacturer’s instructions. Intracellular antibodies are listed in table S1. Stained cells were then analyzed using a BD LSR Fortessa 4L or Cytek Aurora. Flow cytometry data were analyzed using FlowJo (version 10.10.0). Gating strategies for major splenic B and T cell subsets are shown in fig. S6. Surface markers used to define immune cell populations are listed in table S2.

### t-SNE analysis

t-distributed Stochastic Neighbor Embedding (t-SNE) analysis was performed on live, singlet splenocytes. For each mouse, 20,000 cells were randomly downsampled from live, singlet splenocytes using the FlowJo DownSample version 3.3.1 plug-in. The downsampled cells were analyzed by t-SNE in FlowJo. Marker expression profiles used to define cell populations are detailed in table S2. The total numbers of cells contributing to each group’s t-SNE plot were 60,000 for Ctrl (*n* = 3) and 80,000 for Ptpn2 BKO (*n* = 4) mice.

### Immunofluorescence

Mouse spleens were fixed as described (*99*). Briefly, the spleens were fixed in PLP buffer [0.05 M phosphate buffer, 0.1 M l-lysine (pH 7.4), NaIO_4_ (2 mg/ml), and paraformaldehyde (10 mg/ml)] overnight, followed by 30% sucrose. The spleens were then frozen at −80°C in Tissue-Tek OCT and cut into 20-μm sections using a Leica CM3050 S cryostat. The spleen sections were blocked and stained with buffer containing 0.1 M tris, 1% fetal bovine serum, 0.3% Triton X-100, and 1% gelatin from cold water fish scales. Antibodies used are listed in table S1. The images were acquired with a Zeiss LSM880 confocal microscope using a Plan-Apochromat 20x air lens (numerical aperture, 0.8). The MZ thickness was quantified using Fiji. Briefly, a region of interest was generated by tracing the MZ of the splenic follicle. The region of interest was subsequently segmented and filled, and a binary object was generated. The Local Thickness plug-in was then used to determine the thickness of the MZ of each follicle. The average MZ thickness per mouse was used for statistical analyses.

### B cell isolation

Single-cell suspensions were obtained by crushing spleens with a syringe plunger on a 70-μm filter. Cell suspensions were subsequently treated with Lympholyte-M (cat. no. CL5030, Cedarlane) for red blood cell removal following the manufacturer’s instructions. Splenic B cells were isolated using the EasySep Mouse B Cell Isolation Kit (cat. no. 19854, StemCell Technologies) following the manufacturer’s instructions. For MZ B cell isolation, anti-mouse Biotin CD23 (cat. no. 101603, BioLegend) was added to the splenocytes treated with EasySep Mouse B Cell Isolation Cocktail at a concentration of 1:400 and incubated at room temperature for an additional 10 min. The rest of the isolation was performed as described in the manufacturer’s protocol.

### Primary B cell cultures

Isolated B cells were labeled with the CellTrace CFSE Cell Proliferation Kit (cat. no. C34554, Invitrogen) according to the manufacturer’s instructions and subsequently stimulated with F(ab′) fragment anti-mouse IgM (2 or 10 μg/ml; cat. no. 115-006-020, Jackson ImmunoResearch), LPS (10 μg/ml; cat. no. L2880, Sigma-Aldrich), or LPS (10 μg/ml) and IFN-γ (50 ng/ml) (cat. no. 315-05-100UG, PreproTech) for 72 hours. The culture was performed in RPMI 1640 media supplemented with 10% heat-inactivated fetal bovine serum, 1% penicillin/streptomycin, and β-mercaptoethanol (1:1000). B cells were then analyzed via flow cytometry after cell surface and viability staining with Zombie Red (cat. no. 423109, BioLegend) following the manufacturer’s instructions.

### In vitro class-switch recombination

Isolated B cells were cultured with LPS (25 μg/ml) and IL-4 (25 ng/ml) (cat. no. 214-14-100UG, PreproTech) for IgG1. Class-switch recombination was analyzed 72 hours later via flow cytometry. See table S1 for antibodies used.

### Western blot

Resting isolated B cells or isolated B cells stimulated with F(ab′) fragment anti-mouse IgM (cat. no. 115-006-020, Jackson ImmunoResearch) at 10 μg/mL or IFN-γ (cat. no. 315-05-100UG, PreproTech) at 50 ng/ml for 5, 15, or 45 min were harvested, washed with cold phosphate-buffered saline (PBS), and frozen. For Ptpn1/2 inhibitor experiments, isolated wild-type B cells were preincubated with 1 μM KQ-791 (Kanyr Pharma) at room temperature for 1 hour. A noninhibitor control was included in parallel. Cells were then stimulated with anti-IgM as described above, with the inhibitor present in the media during stimulation. Cell pellets were then lysed in modified radioimmunoprecipitation (mRIPA) assay buffer [0.05 M tris (pH 7.4), 0.15 M NaCl, 1% NP-40, and 0.25% sodium deoxycholate] supplemented with 1 mM Na_3_VO_4_ and 5 mM NaF and then subjected to SDS–polyacrylamide gel electrophoresis. Primary antibodies used are listed in table S1. For total protein loading control, we added 0.5% (v/v) tri-chloro-ethanol to acrylamide gel preparations.

### Substrate trapping

Human embryonic kidney (HEK) 293T/17 cells (RRID no. CVCL_1926) were transfected with human 2xHA-LYN and FLAG-PTPN2 wild type or FLAG-PTPN2 D68A trapping mutant. Transfected cells were lysed in immunoprecipitation buffer [0.025 M tris-HCl (pH 7.4), 0.15 M NaCl, 1% Triton X-100, and Protease Inhibitor Cocktail]. Cell lysates were incubated with FLAG magnetic beads for 2 hours at 4°C followed by Western blot analysis.

### In vitro dephosphorylation assay

Recombinant purified His-LYN was incubated with wild-type or D68A mutant recombinant GST-PTPN2 for an hour at 30°C in phosphatase buffer [0.05 M Hepes (pH 7.0) and 3 mM dithiothreitol]. Lambda Protein Phosphatase (cat. no. P0753, New England Biolabs) was used as a positive control for dephosphorylation using the manufacturer’s buffer. Phosphorylation levels of LYN were assessed via Western blotting.

### Mouse immunization

*S. pneumoniae* immunizations were performed by injecting 1 × 10^8^ heat-killed *S. pneumoniae* cells (cat. no. tlrl-hksp, InvivoGen) intravenously via the tail vein. Mice were euthanized, and serum was harvested 5 days postimmunization. Seven female and two male mice were included per genotype in this experiment. These mice represent the combined total from two independent experiments. NP-CGG immunizations were performed by injecting 100 μg of NP(13)-CGG (cat. no. N-5055B, Biosearch Technologies) mixed at a 1:1 ratio with Alhydrogel 2% adjuvant (cat. no. vac-alu-50, InvivoGen) intraperitoneally. Serum was harvested 2 weeks after primary immunization. For recall immunizations, mice were immunized intraperitoneally with 50 μg of NP-CGG and Alhydrogel 2% adjuvant 9 weeks after the primary immunization. Seven female and two male mice per genotype were included in the primary NP-CGG immunization study. These mice represent the combined total from two independent experiments. One female and four male mice per genotype were included in the recall NP-CGG immunization study.

### RNA-seq and analysis

B cells isolated from the spleen of *Ptpn2* BKO (fl/fl) or control mice were stimulated with anti-IgM (10 μg/ml) or IFN-γ (50 ng/ml) ex vivo for 6 hours. Resting B cells for each genotype were also included in this study. Frozen cell pellets were sent to Azenta Life Sciences for RNA extraction and RNA-seq. RNA extraction was done using the RNeasy Plus Universal mini kit (cat. no. 73404, Qiagen) following the manufacturer’s instructions. Library sequencing was performed on an Illumina NovaSeq. The samples were sequenced using a 2 × 150–base pair paired-end configuration. To measure transcript abundance, we quantified the transcripts per million (TPM) for the GENCODE M36 GRCm38 genes using Salmon version 1.9.0 (*100*). Gene-level count matrices were generated using tximport version 1.36.0 (*101*). Differential gene expression between groups of interest was determined using DESeq2 version 1.48.0 (*102*). Gene set enrichment analysis on differentially expressed genes was done using clusterProfiler version 4.16.0 (*103*). Heatmaps were generated using pheatmap version 1.0.12. TF activity inference from differentially expressed genes was done using CollecTRI-derived regulons (*74*) and decoupleR version 2.14.0 (*104*). Volcano plots were generated using ggplot2 version 3.5.2.

### Influenza A virus infection

Mice were infected intranasally with influenza A virus H1N1 strain PR8 (30 PFU/20 g of body weight). Serum and immune organs were collected at day 21 postinfection. For recall infections, mice were infected intranasally with H1N1 PR8 (60 PFU/20 g of body weight) 10 weeks after the primary infection. Euthanasia and analyses were performed 1 week post–recall infection. For primary H1N1 infections, three female and seven male mice per genotype were included. These mice represent the combined total from two independent experiments. For recall H1N1 infections, four female and five male mice were included per genotype. These mice represent the combined total from two independent experiments (*n* = 4 or 5 mice per genotype per experiment).

### ELISA

ELISAs to detect serum antibody isotypes were done using the SBA Clonotyping System-C57BL/6 (cat. no. 5300-05B, Southern Biotech) and the C57BL/6 Mouse Immunoglobulin Panel (cat. no. 5300-01B, Southern Biotech) following the manufacturer’s instructions. ELISAs to detect antigen-specific antibodies in the sera of immunized mice were done by coating 96-well plates with 100 μl of phosphorylcholine-BSA (25 μg/ml; cat. no. PC-1011H-10, Biosearch Technologies), NP(36)-BSA (50 μg/ml; cat. no. N-5050H-100, Biosearch Technologies), NP(2)-BSA (50 μg/ml; cat. no. N-5050XL-10, Biosearch Technologies), CGG (1 μg/ml; cat. no. C-1000, Biosearch Technologies), or heat-inactivated H1N1 viral protein (2.5 μg/ml). Detection was done using HRP (horseradish peroxidase)–conjugated total Ig, IgM, IgG1, IgG2b, IgG2c, or IgG3 (cat. no. 5300-05B, Southern Biotech).

### Relative affinity ELISA

NaSCN displacement H1N1 IgG ELISA was done essentially as described (*105*). Briefly, mouse sera were diluted to a concentration that yielded similar initial absorbance. This was previously determined by H1N1-specific IgG ELISA using Goat Anti-Mouse IgG (Southern Biotech, cat. no. 1030-08) as a detection antibody followed by HRP-Streptavidin (BioLegend, cat. no. 405210). Diluted sera were incubated on 96-well plates coated with heat-inactivated H1N1. After standard washing, plates were treated with a range of NaSCN concentrations (0, 0.0625, 0.125, 0.5, 1, 2, 4, and 5 M) and subsequently blocked with 0.1% porcine gelatin. The rest of the assay was performed following the standard ELISA protocol. Relative affinity values were calculated as described (*105*); briefly, the reduction in signal at each step was multiplied by the corresponding NaSCN concentration (e.g., differences after 0.0625 M, 0.125 M, etc.), and the final residual binding after 5 M NaSCN treatment was multiplied by 6. The sum of these weighted values yields the relative affinity values (RAV), representing the overall antibody affinity for the antigen.

### Microneutralization assay

Sera collected from mice 1 week after recall infection with H1N1 influenza virus were heat inactivated at 65°C for 30 min and serially diluted in a 96-well plate, starting at a 1:40 dilution. Each dilution was incubated with H1N1 virus (100 PFU per 25 μl) for 1 hour at room temperature. The virus-serum mixtures were then added to confluent Madin-Darby canine kidney (MDCK) cell monolayers and incubated for 1 hour at 37°C. Following incubation, cells were washed and overlaid with fresh medium. After 72 hours, cells were fixed with 10% paraformaldehyde and stained with crystal violet to visualize intact monolayers. Neutralizing antibody titers were defined as the reciprocal of the highest serum dilution that completely prevented the virus-induced cytopathic effect.

### Statistics

Statistical analyses were conducted using GraphPad Prism software. For analyses between groups with *n* < 5, a nonparametric test was performed. For analyses between groups with *n* ≥ 5, nonpaired Student’s *t* test was used. For analyses between groups with *n* ≥ 5 in which there was large biological variability resulting from mouse sex and age, paired *t* tests were used. Data distribution was tested using the Shapiro-Wilk test. Error bars represent standard deviation. For antibody titer plots derived from antigen-specific ELISAs in which serum dilution (*x* axis) was plotted against absorbance (*y* axis), two-way analysis of variance (ANOVA) analyses were conducted. Error bars represent the standard error of the mean (SEM). *n* numbers as well as the statistical test used for each figure panel are specified in each figure legend. ns means nonsignificant. *P ≤ 0.05, **P ≤ 0.01, ***P ≤ 0.001, and ****P ≤ 0.0001.
