# Supplementary material for: Ptpn2 limits plasma cell fate and antiviral immunity by integrating B cell receptor and IFN-γ signals in B cells
Source: Sci Adv. 2026 Aug 19;12(34):eaec0162. doi: 10.1126/sciadv.aec0162 (PMC13488884; doi:10.1126/sciadv.aec0162)
Supplement: Supplementary file 1 [file sciadv.aec0162_sm.pdf]

Science Advances
AAAS

Supplementary Materials for

Ptpn2 limits plasma cell fate and antiviral immunity by integrating B cell receptor and IFN-γ signals in B cells

Ana Maria Hincapie *et al.*

Corresponding author: Michel L. Tremblay, michel.tremblay@mcgill.ca

Sci. Adv. **12**, eaec0162 (2026)
DOI: 10.1126/sciadv.aec0162

This PDF file includes:

Figs. S1 to S6
Tables S1 and S2

SUPPLEMENTARY MATERIAL

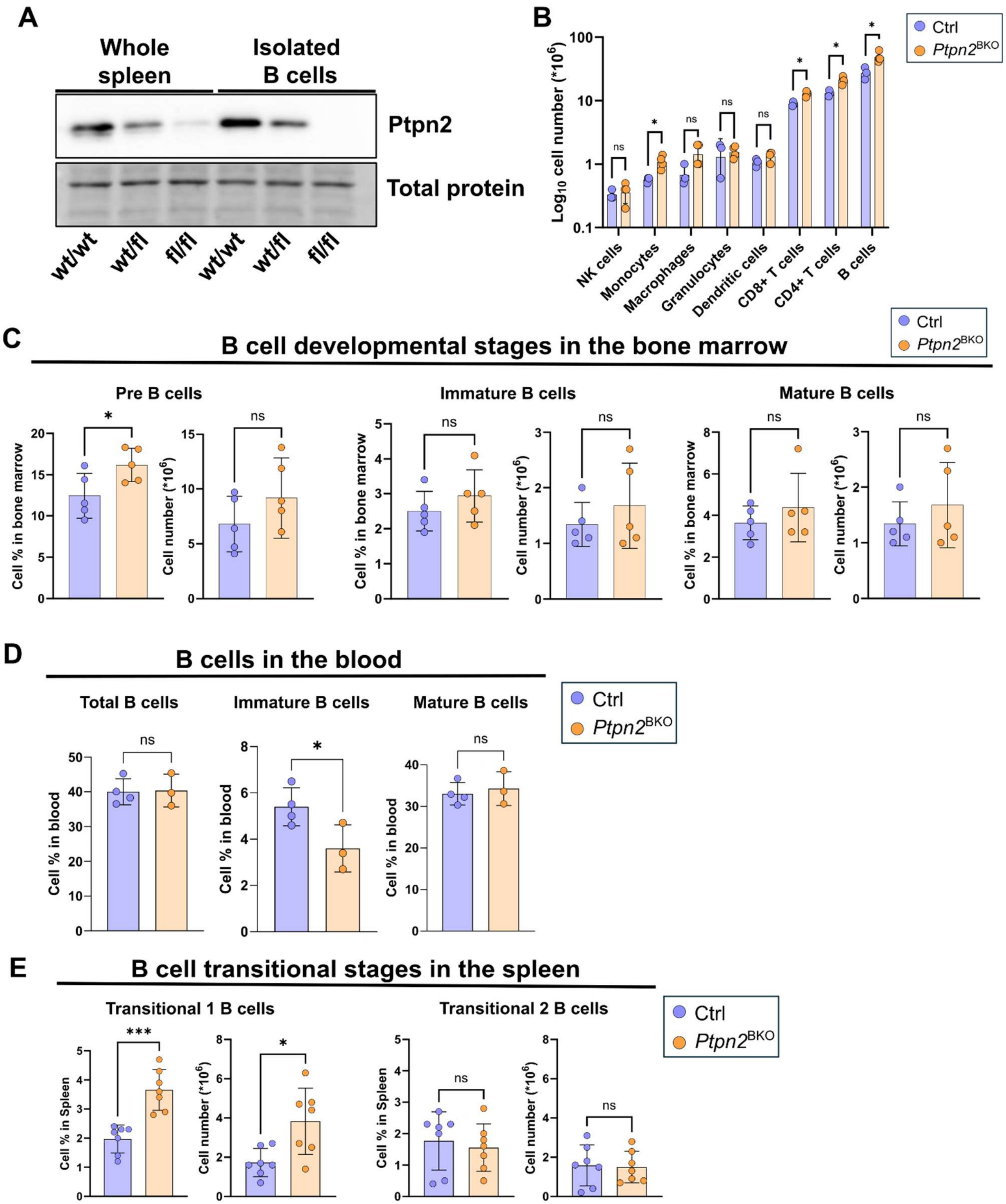

Figure S1. Characterization of Ptpn2 B-cell-specific knockout mice. All panels compare $Ptpn2^{fl/fl}$MB1-cre (*Ptpn2*^{BKO}) versus $Ptpn2^{wt/wt}$MB1-cre (Ctrl) mice **A)** Immunoblot showing Ptpn2 expression in splenocytes or isolated B cells from the spleen of $Ptpn2^{wt/wt}$MB1-cre (wt/wt), $Ptpn2^{fl/wt}$MB1-cre (fl/wt) and $Ptpn2^{fl/fl}$MB1-cre (fl/fl) mice using a negative selection kit (CD11b-, CD4-, CD8a-, Ly6G/C-, Ter119-, CD43-, CD49b-, and CD90.2- fraction). Total protein was used as a loading control **B)** Absolute numbers of splenic immune cell subsets. Numbers were Log10 transformed for visualization. Data represent one out of at least two independent experiments **C)** Frequencies and absolute numbers of CD19+ B220+CD93+CD43-IgM-IgD- pre B cells, CD19+B220+CD93+IgM+IgD- immature B cells and CD19+B220+CD93-IgM+IgD+ mature B cells in the bone marrow of mice of the indicated genotypes. Data represent one out of two independent experiments **D)** Frequencies of CD19+B220+ total B cells, CD19+B220+CD3-CD93+ transitional 1 B cells and CD19+B220+CD3-CD93-IgM^{lo}IgD+ mature B cells in the blood of mice of the indicated genotypes. Data represent one out of two independent experiments **E)** Percentages and absolute numbers of CD19+B220+CD93+IgM+CD23- transitional 1 B cells and CD19+B220+CD93+IgM+CD23+ transitional 2 B cells in the spleen of mice of the indicated genotypes. Data represent pooled results from two independent experiments. Statistical analyses were done using non-parametric test (B, D), unpaired t test (C, E). ns, non-significant. $*p \leq 0.05$, $**p \leq 0.01$, $***p \leq 0.001$, $****p \leq 0.0001$. Each dot represents a biological replicate.

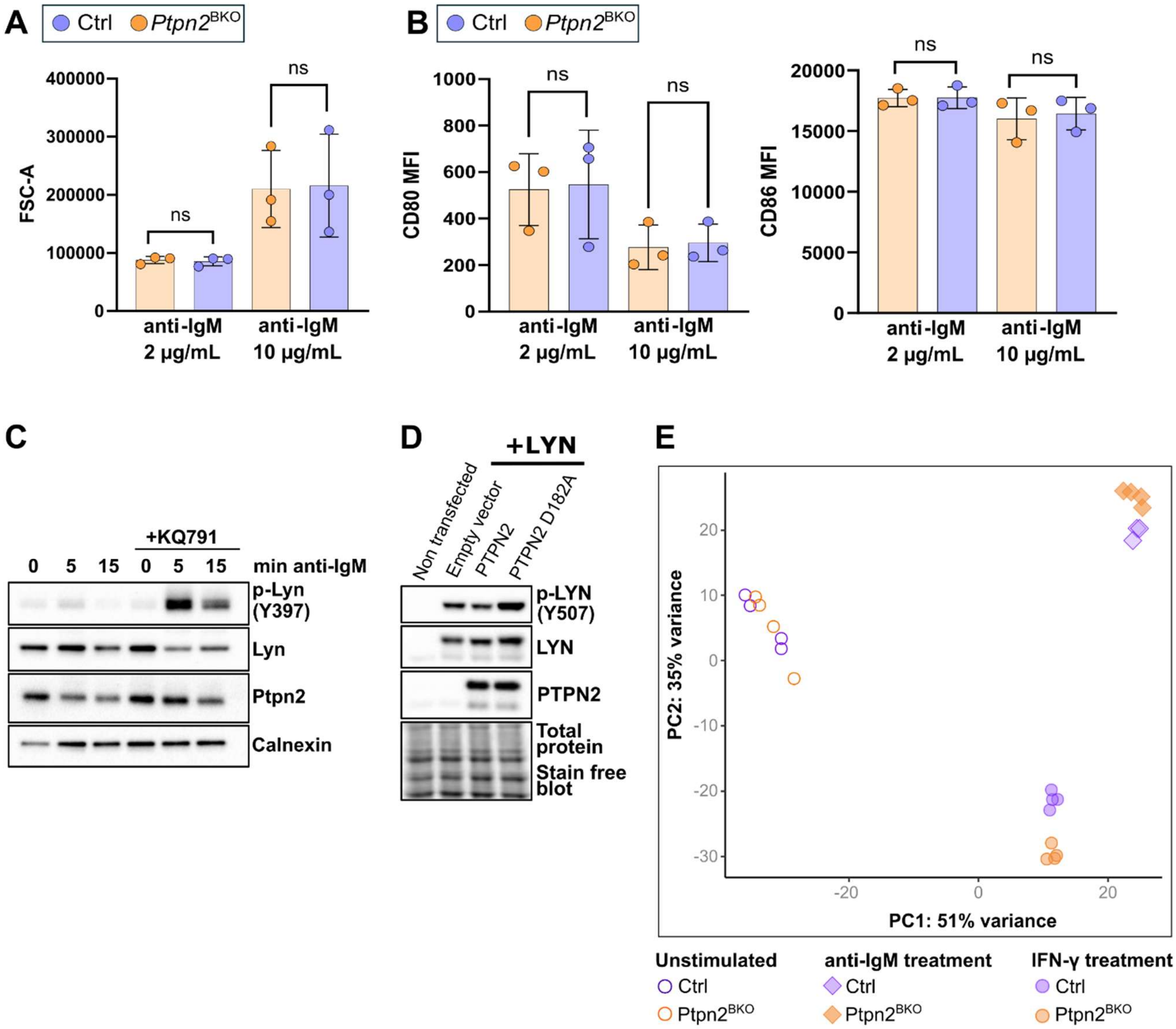

A
Ctrl
Ptpn2BKO
FSC-A
ns
anti-IgM 2 µg/mL
anti-IgM 10 µg/mL
B
CD80 MFI
CD86 MFI
C
+KQ791
0 5 15 0 5 15 min anti-IgM
p-Lyn (Y397)
Lyn
Ptpn2
Calnexin
D
+LYN
Non transfected
Empty vector
PTPN2
PTPN2 D182A
p-LYN (Y507)
LYN
PTPN2
Total protein
Stain free blot
E
PC1: 51% variance
PC2: 35% variance
Unstimulated
anti-IgM treatment
IFN-γ treatment
Ctrl
Ptpn2BKO

Figure S2. Ptpn2-deficient B cells display normal in vitro activation in response to anti-IgM stimulation. All panels compare Ptpn2$^{fl/fl}$MB1-cre (*Ptpn2*^{BKO}) versus Ptpn2$^{wt/wt}$MB1-cre (Ctrl) mice **A)** Cell size quantified via flow cytometry using forward size scatter (FSC-A) of isolated B cells after a 72-hour incubation with 2 or 10 µg/mL anti-IgM **B)** Expression levels of CD80 and CD86 in isolated B cells after a 72-hour incubation with 2 or 10 µg/mL anti-IgM. Surface expression is quantified via flow cytometry using mean fluorescence intensity (MFI) **C)** Immunoblot analysis of phosphorylated (p) and total Lyn Y397 of splenic B cells isolated from wild-type (Ptpn2$^{wt/wt}$ Cre$^{-/-}$) mice with or without treatment with Ptpn2 inhibitor (KQ791) at baseline, 5 or 15 minutes of anti-IgM stimulation. Data is representative of two independent experiments **D)** HEK293T/17 cells were transfected with HA-LYN and vector control or constructs for wild-type or D182A PTPN2. p-LYN at position Y507 was analyzed via immunoblotting. Data is representative of three independent experiments **E)** PCA of DESeq2-normalized bulk RNA-seq data from isolated mouse total splenic B cells under the indicated conditions. PC1 and PC2 explain 52% and 35% of the variance, respectively. Statistical analyses are done using non-parametric test (A, B). ns, non-significant. $*p \leq 0.05$, $**p \leq 0.01$, $***p \leq 0.001$, $****p \leq 0.0001$. Each dot represents a biological replicate.

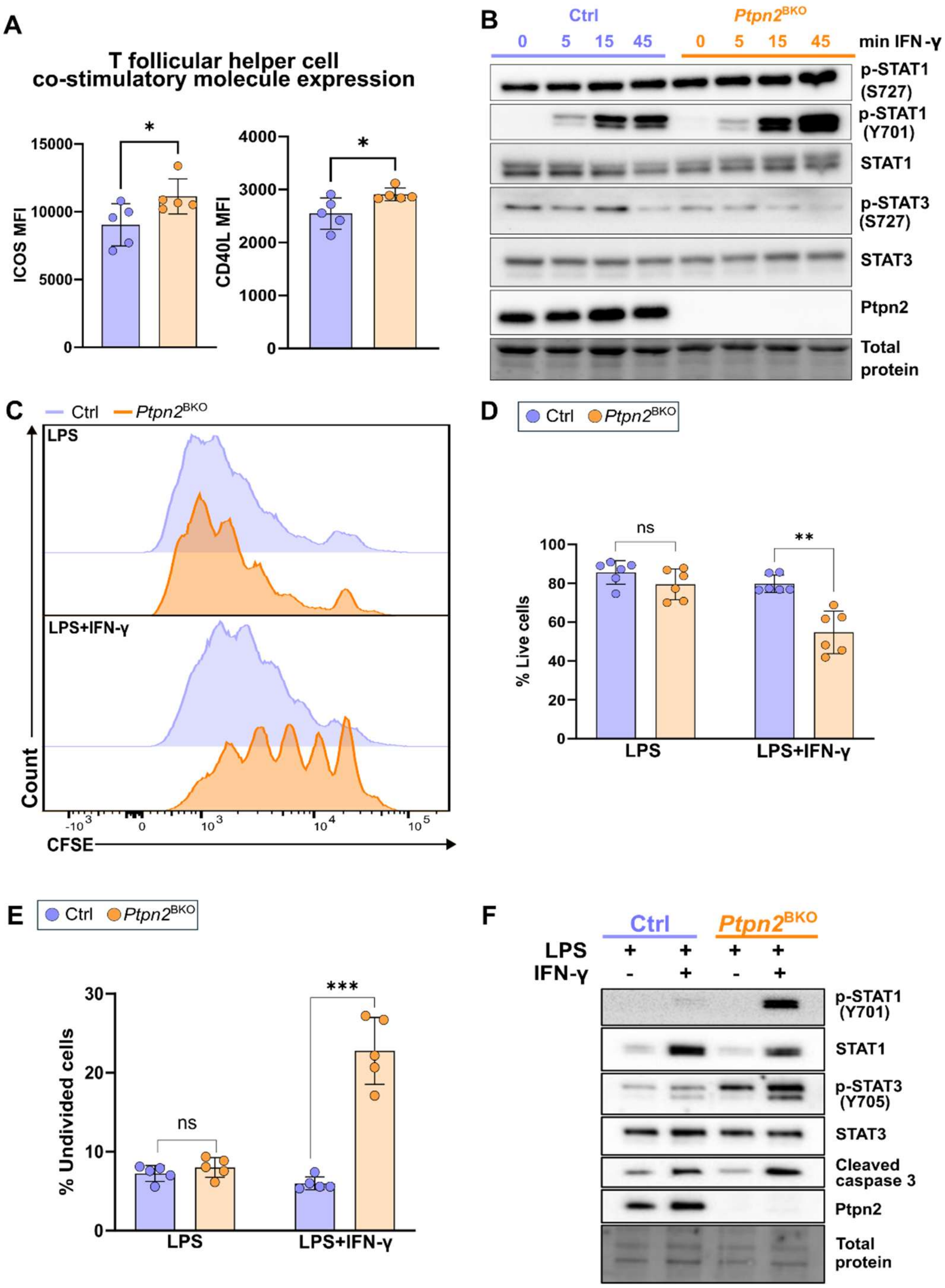
A
T follicular helper cell co-stimulatory molecule expression
ICOS MFI
CD40L MFI
B
Ctrl
Ptpn2BKO
0 5 15 45 0 5 15 45 min IFN-γ
p-STAT1 (S727)
p-STAT1 (Y701)
STAT1
p-STAT3 (S727)
STAT3
Ptpn2
Total protein
C
Ctrl Ptpn2BKO
LPS
LPS+IFN-γ
Count
CFSE
D
Ctrl Ptpn2BKO
% Live cells
LPS
LPS+IFN-γ
E
Ctrl Ptpn2BKO
% Undivided cells
LPS
LPS+IFN-γ
F
Ctrl
Ptpn2BKO
LPS
IFN-γ
p-STAT1 (Y701)
STAT1
p-STAT3 (Y705)
STAT3
Cleaved caspase 3
Ptpn2
Total protein

Figure S3. Ptpn2-deficient B cells display decreased survival and delayed proliferation due to sustained STAT1 activation in vitro. All panels compare $Ptpn2^{fl/fl}$MB1-cre (*Ptpn2*^{BKO}) versus $Ptpn2^{wt/wt}$MB1-cre (Ctrl) mice **A)** Mean fluorescence intensity (MFI) of ICOS and CD40L in splenic Tfh cells. **B)** Immunoblot analysis of phosphorylated (p) and total STAT1 Y727 and Y701, and STAT3 Y727 of isolated splenic B cells at baseline, 5, 15 or 45 minutes of stimulation with IFN-γ. Data is representative of two independent experiments. **C)** Flow cytometric analysis of CFSE proliferation histograms of B cells isolated from spleens of mice of the indicated genotypes stimulated ex vivo for 72 hours with LPS or LPS and IFN-γ **D)** Percentages of live B cells after a 72-hour incubation with 10 μg/mL LPS or 10 μg/mL LPS and 50 ng/mL IFN-γ. Live cells are quantified via flow cytometry using a viability dye. Data represent pooled results from two independent experiments **E)** Percentages of undivided B cells after a 72-hour incubation with 10 μg/mL LPS or 10 μg/mL LPS and 50 ng/mL IFN-γ. Undivided cells are quantified via flow cytometry using CFSE as a proliferation marker. Data represent pooled results from two independent experiments **F**) Immunoblot analysis of phosphorylated (p) and total STAT1 and STAT3 as well as cleaved caspase 3 of B cells isolated from spleens of mice of the indicated genotypes after a 72-hour incubation with 10 μg/mL LPS or 10 μg/mL LPS and 50 ng/mL IFN-γ. Data is representative of three independent experiments. Statistical analyses for all figures are done using unpaired t test. ns, non-significant. $^*p \leq 0.05$, $^{**}p \leq 0.01$, $^{***}p \leq 0.001$, $^{****}p \leq 0.0001$. Each dot represents a biological replicate.

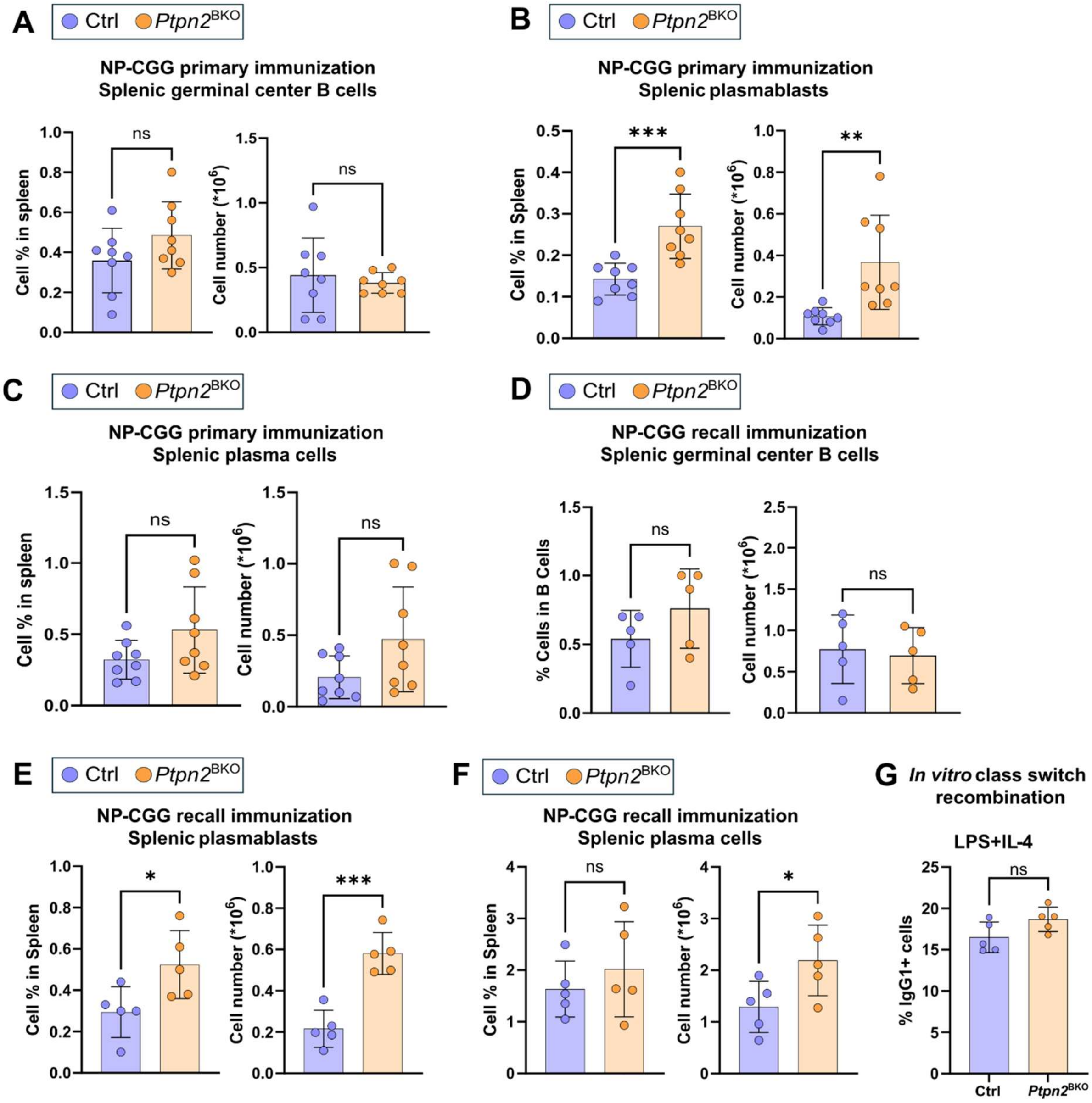
A
Ctrl
Ptpn2BKO
NP-CGG primary immunization
Splenic germinal center B cells
Cell % in spleen
Cell number (*10^6)
ns
B
NP-CGG primary immunization
Splenic plasmablasts
Cell % in Spleen
Cell number (*10^6)
***
**
C
NP-CGG primary immunization
Splenic plasma cells
D
NP-CGG recall immunization
Splenic germinal center B cells
% Cells in B Cells
E
NP-CGG recall immunization
Splenic plasmablasts
*
F
NP-CGG recall immunization
Splenic plasma cells
G
In vitro class switch recombination
LPS+IL-4
% IgG1+ cells

Figure S4. Characterization of B cell responses to primary and recall immunization of Ptpn2 B-cell-specific knockout mice. All panels compare Ptpn2$^{fl/fl}$MB1-cre (*Ptpn2*^{BKO}) versus Ptpn2$^{wt/wt}$MB1-cre (Ctrl) mice **A-C)** Frequencies and absolute cell numbers of splenic (A) germinal center B cells, (B) plasmablasts and (C) plasma cells in mice of the indicated genotypes 15 days post primary immunization with NP-CGG. Data is representative of two pooled independent experiments **D-F)** Frequencies and absolute cell numbers of splenic (D) germinal center B cells, (E) plasmablasts and (F) plasma cells in mice of the indicated genotypes 7 days post recall immunization with NP-CGG **G)** B cells were isolated from the spleens of uninfected mice of the indicated genotypes and stimulated with 25 µg/mL LPS and 50 ng/mL IL-4 for 72 hours. Class switch recombination to IgG1 was assessed via flow cytometry. Data represent pooled results from two independent experiments. Statistical analyses are done using non-parametric test (A, B, C) and unpaired t test (D, E, F, G). ns, non-significant. *$p \leq 0.05$, **$p \leq 0.01$, ***$p \leq 0.001$, ****$p \leq 0.0001$. Each dot represents a biological replicate.

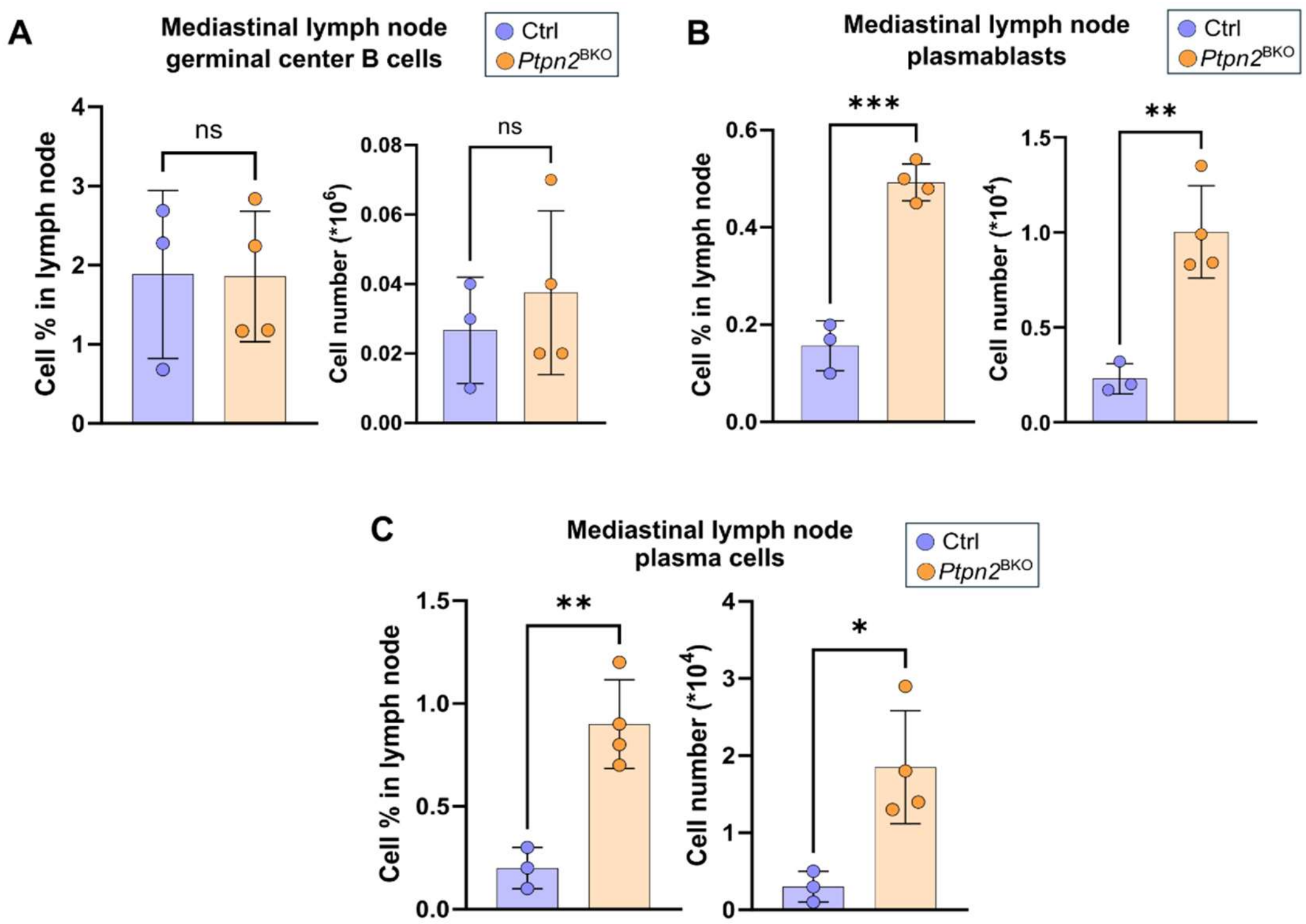
A
Mediastinal lymph node
germinal center B cells
Ctrl
Ptpn2BKO
ns
Cell % in lymph node
0
1
2
3
4
ns
Cell number (*10^6)
0.00
0.02
0.04
0.06
0.08
B
Mediastinal lymph node
plasmablasts
Ctrl
Ptpn2BKO
***
Cell % in lymph node
0.0
0.2
0.4
0.6
**
Cell number (*10^4)
0.0
0.5
1.0
1.5
C
Mediastinal lymph node
plasma cells
Ctrl
Ptpn2BKO
**
Cell % in lymph node
0.0
0.5
1.0
1.5
*
Cell number (*10^4)
0
1
2
3
4

Figure S5. Analysis of B cell subsets in the mediastinal lymph node following influenza H1N1 infection. All panels compare $Ptpn2^{fl/fl}$MB1-cre (*Ptpn2*^{BKO}) versus $Ptpn2^{wt/wt}$MB1-cre (Ctrl) mice **A-C)** Frequencies and absolute cell numbers of (A) germinal center B cells, (B) plasmablasts and (C) plasma cells in the mediastinal (lung draining) lymph node of mice of the indicated genotypes 7 days post recall infection with influenza H1N1. Statistical analyses for all figures are done using unpaired t test. ns, non-significant. $^*p \leq 0.05$, $^{**}p \leq 0.01$, $^{***}p \leq 0.001$, $^{****}p \leq 0.0001$. Each dot represents a biological replicate.

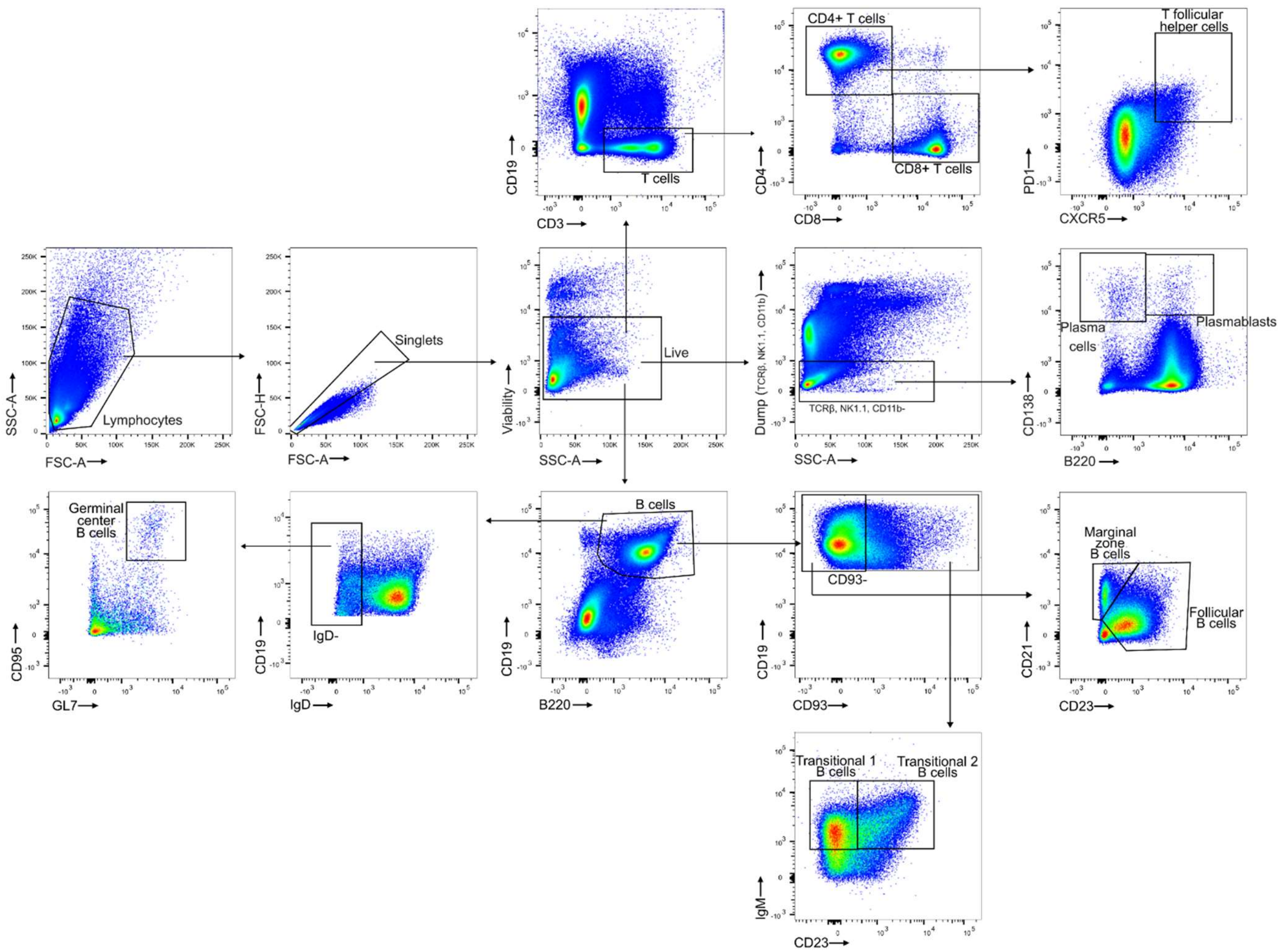

Lymphocytes
SSC-A
FSC-A
Singlets
FSC-H
FSC-A
Live
Viability
SSC-A
T cells
CD19
CD3
CD4+ T cells
CD8+ T cells
CD4
CD8
T follicular helper cells
PD1
CXCR5
Dump (TCRβ, NK1.1, CD11b)
TCRβ, NK1.1, CD11b-
SSC-A
Plasma cells
Plasmablasts
CD138
B220
B cells
CD19
B220
IgD-
CD19
IgD
Germinal center B cells
CD95
GL7
CD93-
CD19
CD93
Marginal zone B cells
Follicular B cells
CD21
CD23
Transitional 1 B cells
Transitional 2 B cells
IgM
CD23

Figure S6: Gating strategy of splenic lymphocyte populations. Representative flow cytometry plots showing the sequential gating strategy used to identify lymphocyte populations. Splenocytes were first gated based on forward- and side-scatter properties (FSC-A vs. SSC-A), followed by exclusion of doublets (FSC-A vs. FSC-H) and dead cells using a viability dye. Specific B and T cell populations were identified as shown.

Table S1: Resource for antibodies

Reagent	Source	Catalogue #
**Cell surface antibodies**		
AF647 CD19 (Mouse)	Biolegend	115522
APC-Cy7 CD19 (Mouse)	Biolegend	115530
BV421 CD19 (Mouse)	Biolegend	115537
AF700 B220 (Mouse/Human)	Biolegend	103232
PerCP-Cy5.5 B220 (Mouse/Human)	Biolegend	103236
BV605 IgM (Mouse)	Biolegend	406523
FITC IgD (Mouse)	Biolegend	405703
APC TCR-β chain (Mouse)	Biolegend	109212
PE-Dazzle594 CD4 (Mouse)	Biolegend	100455
Pacific Blue CD8 (Mouse)	Biolegend	100725
BV421 CD44 (Mouse)	Biolegend	103039
CD62L CD62L (Mouse)	BD Biosciences	564109
FITC NK1.1 (Mouse)	Biolegend	108705
PE CD49b (Mouse)	Biolegend	108907
PE-Cy7 Ly-6G (Mouse)	Biolegend	127617
APC-Cy7 Ly-6C(Mouse)	BD Biosciences	560596
Spark YG 593 CD11b (Mouse/Human)	Biolegend	101281
BV510 CD11c (Mouse)	Biolegend	117337
PE-Cy5 F4/80 (Mouse)	Biolegend	123111
Spark Blue 550 I-A/I-E (Mouse)	Biolegend	107661
APC-Cy7 CD21/35 (Mouse)	Biolegend	123417
AF700 CD23 (Mouse)	Biolegend	101632
FITC CD23 (Mouse)	Biolegend	101605
PE-Cy7 CD93 (Mouse)	Biolegend	136505
PE CD43 (Mouse)	BD Biosciences	553271
APC CD24 (Mouse)	Biolegend	138505
PE-Cy7 CD80 (Mouse)	Biolegend	104733
BV785 CD86 (Mouse)	Biolegend	105043
APC CD95 (Mouse)	Biolegend	152603
PE GL7 (Mouse)	Biolegend	144605
PE CD138 (Mouse)	Biolegend	142503
PerCP-Cy5.5 PD1 (Mouse)	Biolegend	109119
APC PD1 (Mouse)	Biolegend	109111
BV421 CXCR5 (Mouse)	Biolegend	145511
PE-Cy7 ICOS (Mouse	Biolegend	117422
APC CD40L (Mouse)	Biolegend	106509
PE IgG1 (Mouse)	BD Pharmingen	550083

**Intracellular antibodies**		
AF700 IFN-γ (Mouse)	Biolegend	505823
AF647 Blimp1 (Mouse)	Biolegend	150004
**Immunofluorescence antibodies**		
AF647 IgM (Mouse)	Biolegend	406526
FITC IgD (Mouse)	Biolegend	405703
BV421 CD169 (Mouse)	Biolegend	142421
**Western blot antibodies**		
Ptpn2	Generated in-house	Clone 3E2
Phospho-PLCγ2 (Y1217)	Cell Signaling Technology	3871
PLCγ2	Cell Signaling Technology	3872
Phospho-Lyn (Y397)	Cell Signaling Technology	70926
Lyn	Cell Signaling Technology	2796
Phospho-Nf-Kb (S536)	Cell Signaling Technology	3033
Nf-κB	Cell Signaling Technology	8242
Phospho-Lyn (Y507)	Cell Signaling Technology	2731
Phospho-STAT1 (Y701)	Cell Signaling Technology	9167
Phospho-STAT1 (Y727)	Cell Signaling Technology	8826
STAT1	Cell Signaling Technology	9172
Phospho-STAT3 (Y705)	Cell Signaling Technology	9145
Phospho-STAT3 (Y727)	Cell Signaling Technology	9134
STAT3	Cell Signaling Technology	4904
Cleaved Caspase 3	Cell Signaling Technology	9661
Calnexin	Generated in-house	Dr. John Bergeron

Table S2: Surface marker definitions for immune cell populations analyzed by flow cytometry

Cell population	Tissue	Markers
Parent gate	All	FSC/SSC → singlets → live cells
Pre B cells	Bone marrow	B220+CD19+CD93+CD43-IgM-IgD-
Immature B cells	Bone marrow	CD19+B220+CD93+IgM+IgD-
Mature B cells	Bone marrow	CD19+B220+CD93-IgM+IgD+
Transitional 1 /Immature B cells	Blood	CD19+B220+CD3-CD93+
Mature B cells	Blood	CD19+B220+CD3-CD93-IgM^{lo}IgD+
B cells	Blood, spleen	CD19+B220+
Transitional 1 B cells	Spleen	CD19+B220+CD93+IgM+CD23-
Transitional 2 B cells	Spleen	CD19+B220+CD93+IgM+CD23+
Marginal zone B cells	Spleen	CD19+B220+CD93-CD21/35^{hi}CD23^{lo}
Follicular B cells	Spleen	CD19+B220+CD93-CD21/35^{lo}CD23^{hi}
Germinal center B cells	Spleen, lymph nodes	CD19+IgD-CD95+GL7+
Plasmablasts	Spleen, lymph nodes	TCRβ-NK1.1-CD11b-B220^{int}CD138+
Plasma cells	Spleen, lymph nodes	TCRβ-NK1.1-CD11b-B220^{lo}CD138+
T cells	Spleen	CD19-CD3+
CD4+ T cells	Spleen	CD19-CD3+CD4+CD8-
CD8+ T cells	Spleen	CD19-CD3+CD4-CD8+
T follicular helper cells	Spleen	CD19-CD3+CD4+CXCR5+PD1+
Dendritic cells	Spleen	CD19-B220-CD3-NK1.1-Ly6G-CD11c+MHCII+
Granulocytes	Spleen	Ly6G+SSC-A^{hi}
Macrophages	Spleen	CD19-B220-CD3-NK1.1-Ly6G-CD11b+Ly6C-F4/80^{hi}
Monocytes	Spleen	CD19-B220-CD3-NK1.1-Ly6G-CD11b+Ly6C^{hi}F4/80^{lo}
Natural killer cells	Spleen	CD19-B220-CD3-NK1.1+CD49b+
